# In Silico Insights: QSAR Modeling of TBK1 Kinase Inhibitors for Enhanced Drug Discovery

**DOI:** 10.1021/acs.jcim.4c00864

**Published:** 2024-09-17

**Authors:** Julian M. Ivanov, Rumiana Tenchov, Krittika Ralhan, Kavita A. Iyer, Shivangi Agarwal, Qiongqiong Angela Zhou

**Affiliations:** † CAS, A Division of the American Chemical Society, Columbus, Ohio 43210, United States; ‡ ACS International India Pvt. Ltd., Pune 411044, India

## Abstract

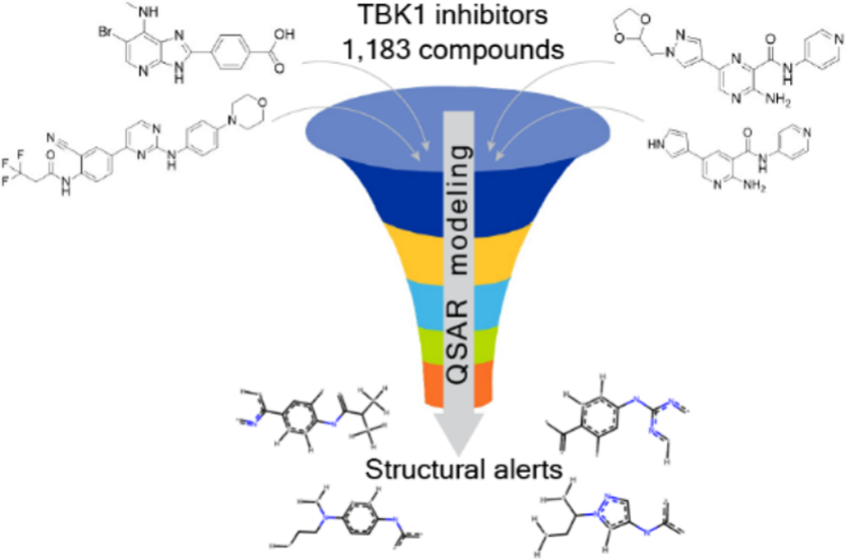

TBK1, or TANK-binding kinase 1, is an enzyme that functions as a serine/threonine protein kinase. It plays a crucial role in various cellular processes, including the innate immune response to viruses, cell proliferation, apoptosis, autophagy, and antitumor immunity. Dysregulation of TBK1 activity can lead to autoimmune diseases, neurodegenerative disorders, and cancer. Due to its central role in these critical pathways, TBK1 is a significant focus of research for therapeutic drug development. In this paper, we explore data from the CAS Content Collection regarding TBK1 and its implication in a large assortment of diseases and disorders. With the demand for developing efficient TBK1 inhibitors being outlined, we focus on utilizing a machine learning approach for developing predictive models for TBK1 inhibition, derived from the fragment-functional analysis descriptors. Using the extensive CAS Content Collection, we assembled a training set of TBK1 inhibitors with experimentally measured IC50 values. We explored several machine learning techniques combined with various molecular descriptors to derive and select the best TBK1 inhibitor QSAR models. Certain significant structural alerts that potentially contribute to inhibition of TBK1 are outlined and discussed. The merit of the article stems from identifying the most adequate TBK1 QSAR models and subsequent successful development of advanced positive training data to facilitate and enhance drug discovery for an important therapeutic target such as TBK1 inhibitors, based on an extensive, wide-ranging set of scientific information provided by the CAS Content Collection.

## Introduction

Kinases are enzymes that catalyze the transfer of a phosphate group from a high-energy molecule, such as adenosine triphosphate (ATP), to a specific substrate molecule, typically a protein, lipid, or carbohydrate.^1^ This process is known as phosphorylation and plays a crucial role in cellular signaling pathways, regulating various cellular functions including metabolism, growth, differentiation, and cell death.^2^ Kinases are essential for transmitting signals within cells and coordinating cellular responses to extracellular stimuli. They are involved in a wide range of physiological processes and are often targets for drug development in treating diseases such as cancer and inflammatory disorders.^3^ Tank-binding kinase 1 (TBK1) is one such enzyme with kinase activity. Encoded by the TBK1 gene in humans, it is a pivotal serine/threonine kinase that orchestrates a variety of critical cellular processes, including innate immunity, inflammation, autophagy, and cell survival/proliferation.^4−12^

Since its discovery, TBK1 has emerged as a central node in the signaling pathways that underpin the defense mechanisms of the body against pathogens and maintain cellular homeostasis. Its ability to phosphorylate and activate key transcription factors, such as interferon regulatory factors (IRFs) and nuclear factor kappa-light-chain-enhancer of activated B cells (NF-κB), underscores its essential role in immune responses.^13−15^

The involvement of TBK1 in immune signaling begins with its activation by pattern recognition receptors (PRRs), which detect pathogen-associated molecular patterns (PAMPs) and damage-associated molecular patterns (DAMPs).^16−18^ Upon activation, TBK1 phosphorylates downstream effectors to induce the expression of type I interferons and pro-inflammatory cytokines, which are crucial for antiviral defense and the modulation of inflammatory responses. Furthermore, TBK1 is integral to the autophagy pathway, where it regulates the selective degradation of ubiquitinated proteins and damaged organelles, thereby contributing to cellular quality control and stress responses.^19−21^

The significance of TBK1 extends beyond normal physiological functions; its dysregulation is implicated in a range of pathological conditions. Mutations and altered expression of TBK1 have been associated with neurodegenerative diseases such as amyotrophic lateral sclerosis (ALS) and frontotemporal dementia (FTD), highlighting its role in neuronal homeostasis. Additionally, TBK1’s involvement in oncogenic pathways links it to cancer progression and survival, making it a potential target for therapeutic intervention.^22,23^

Given its central role in multiple signaling pathways, TBK1 represents a critical juncture in the regulation of immune responses, inflammation, and cell survival. This paper aims to provide a comprehensive overview of TBK1’s structural features, its regulatory mechanisms, and its diverse functional roles in health and disease. By elucidating the molecular intricacies of TBK1, we can better understand its contributions to cellular homeostasis and its potential as a therapeutic target in various disease contexts. Its intricate functions make it an intriguing target for further research and therapeutic interventions.^22,24−27^

The objective of this article is to utilize machine learning approach for developing valuable predictive models for TBK1 inhibition–a topic of utmost importance as an attractive target for drug development. We explored data from the CAS Content Collection,^28^ the world’s largest human expert-curated collection of scientific data, regarding TBK1 and its implication in a large assortment of diseases and disorders. With the demand for developing efficient TBK1 inhibitors outlined, we further focused on developing predictive analytics for TBK1 inhibition, derived from the fragment-functional analysis descriptors. For such fragment-based methodology, the molecular descriptors are the structural alerts obtained by splitting the chemical structures of the training set into all possible subfragments. Based on data from the CAS Content Collection, we assembled a training set of known TBK1 inhibitors whose IC50 values have been determined experimentally. We explored several machine learning techniques combined with various molecular descriptors to derive and select the best TBK1 QSAR models. We also considered various aspects of TBK1 structure and potential inhibitors. Certain significant structural alerts potentially important for TBK1 inhibition have been outlined and discussed. The novelty and merit of the article stem from identifying the most adequate TBK1 QSAR models and subsequent successful development of advanced positive training data to facilitate and enhance drug discovery for an important therapeutic target such as TBK1, based on extensive, wide-ranging set of scientific information provided by the CAS Content Collection.

## TBK1 Overview and Landscape of Research Progress

### Importance of TBK1 in Cellular Processes

TBK1 is a multifunctional protein kinase that plays a crucial role in various cellular processes.^14,29−32^

- TBK1 is a key regulator of the innate immune response to viral and bacterial infections. It is activated upon detection of viral nucleic acids by pattern recognition receptors (PRRs) in the cytoplasm. Activated TBK1 phosphorylates the transcription factor IRF3 (interferon regulatory factor 3), leading to its dimerization and translocation to the nucleus. IRF3 induces the expression of type I interferons and other antiviral genes, which help to limit viral replication and spread.^33,34^
- TBK1 is involved in the regulation of inflammatory responses.^7,8^ It can activate the transcription factor NF-κB (nuclear factor kappa-light-chain-enhancer of activated B cells) by phosphorylating IκBα (inhibitor of NF-κB α), leading to its degradation and the subsequent release of NF-κB. NF-κB translocates to the nucleus and induces the expression of pro-inflammatory cytokines, chemokines, and other inflammatory mediators.
- TBK1 plays a role in the regulation of autophagy, a cellular process involved in the degradation and recycling of damaged organelles and proteins.^9,10^ TBK1 phosphorylates autophagy-related proteins such as ULK1 (Unc-51 like autophagy activating kinase 1) and OPTN (optineurin), promoting autophagosome formation and maturation.
- TBK1 signaling contributes to cell survival and proliferation in various contexts. It can activate the AKT (protein kinase B) pathway, which promotes cell survival and growth, and it regulates the expression of antiapoptotic genes.^35,36^
- TBK1 is involved in the regulation of metabolic processes, including glucose metabolism and lipid homeostasis.^37−39^ It can modulate insulin signaling pathways and influence the expression of genes involved in metabolism.
- TBK1 has been implicated in the cellular response to DNA damage.^27,40^ It can phosphorylate and activate the DNA repair protein BRCA1 (breast cancer type 1 susceptibility protein), contributing to the repair of DNA double-strand breaks.

### TBK 1 Structure

TBK1 is a noncanonical IKK kinase, which phosphorylates the nuclear factor kB. Consisting of 729 amino acids, TBK1 includes four main domains: an N-terminal kinase domain (KD; 1-307), an ubiquitin-like domain (ULD; 308-384), and two coiled-coil domains (CCD1; 407-657 and CCD2; 659-713).^41^

The KD is responsible for the catalytic activity of TBK1. It adopts a typical protein kinase fold and contains the active site for phosphorylation reactions. The ULD, located adjacent to the KD, plays a role in regulating TBK1 activity and interactions. The CCD1, also referred to as scaffold dimerization domain (SDD), harbors a leucine zipper domain (LZ; 499-527) and a helix–loop–helix domain (HLH; 591-632), both of which mediate dimerization of TBK1 molecules. It forms extensive interactions with both the KD and ULD, contributing to the overall stability of the TBK1 structure.^42^ The CCD2 at the C-terminus holds an adaptor-binding motif which assists the interaction of TBK1 with adaptor proteins, such as TANK, NAK–associated protein, TBKBP1, or optineurin.^42−46^ TBK1 forms an intimate dimer through extensive interactions between the SDDs, KDs, and ULDs of two TBK1 molecules. This dimerization is crucial for TBK1 function and regulation.^30,41,43,47−49^

The various domains of TBK1 allow it to participate in multiple cellular processes, including: (i) immune response: TBK1 phosphorylates and activates IRF3 and IRF7, leading to the production of type I interferons in response to viral infection; (ii) NF-κB pathway: TBK1 can activate the NF-κB pathway, promoting the expression of pro-inflammatory cytokines; (iii) autophagy: by phosphorylating autophagy-related proteins, TBK1 regulates the degradation of cellular components, which is important for cellular homeostasis and defense against pathogens; (iv) cell proliferation and survival: TBK1 is involved in pathways that control cell growth and survival, linking it to cancer biology.^41,43,48,49^

**Figure 1:**
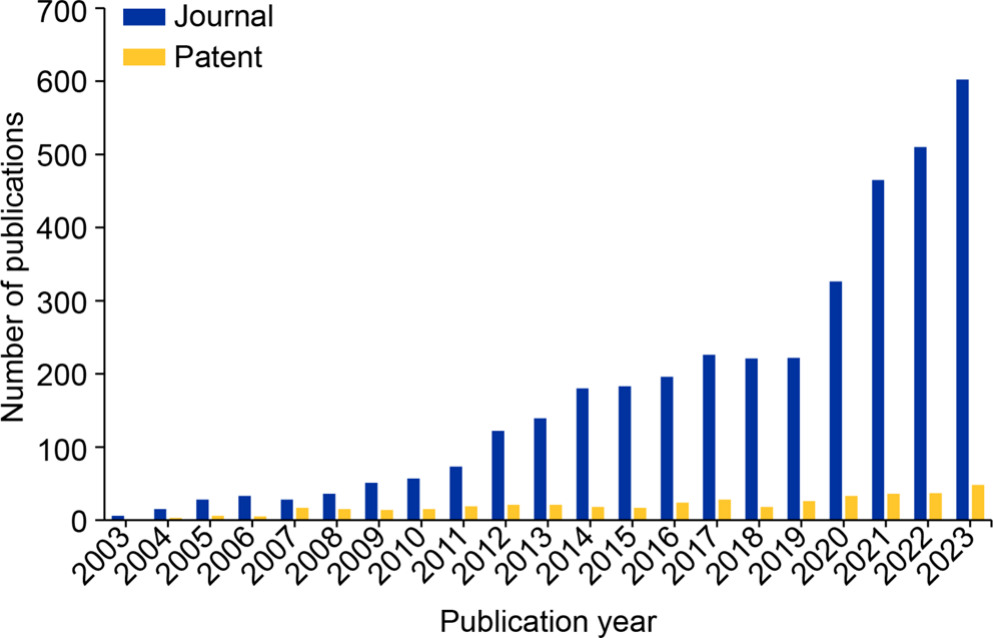
Publications related to TBK1 from the CAS Content Collection for the period 2003–2023.

Journal publications related to TBK1 have grown rapidly and consistently over the last two decades, nearly doubling between 2020 and 2023 as seen from data leveraged from the CAS Content Collection.^28^ This active and rapid increase is indicative of interest in TBK1 from the scientific community. Growth in patent publications on the other hand has been slow, speaking to potential difficulties in targeting TBK1 (elaborated briefly in later sections). The journal-to-patent ratio was >12X for the year 2023 (Figure 1).

**Figure 2:**
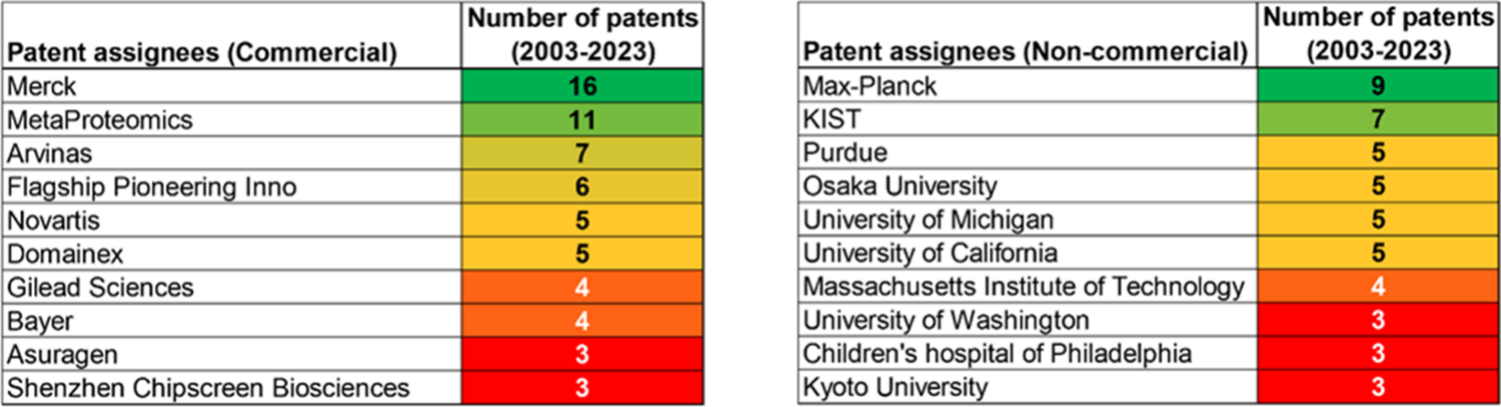
Leading patent assignees in the field of TBK1-related research based on data from CAS Content Collection for the period 2003–2023. Patent assignees have been separated into two categories–commercial (left panel) and noncommercial (right panel).

Patenting activity is dominated by corporate players as compared to academics (Figure 2). Merck, MetaProteomics, and Arvinas have the highest number of patent applications among commercial entities, while Max-Planck Institute, the Korea Institute of Science and Technology (KIST), and Purdue University are the leaders among the academic organizations.

**Figure 3:**
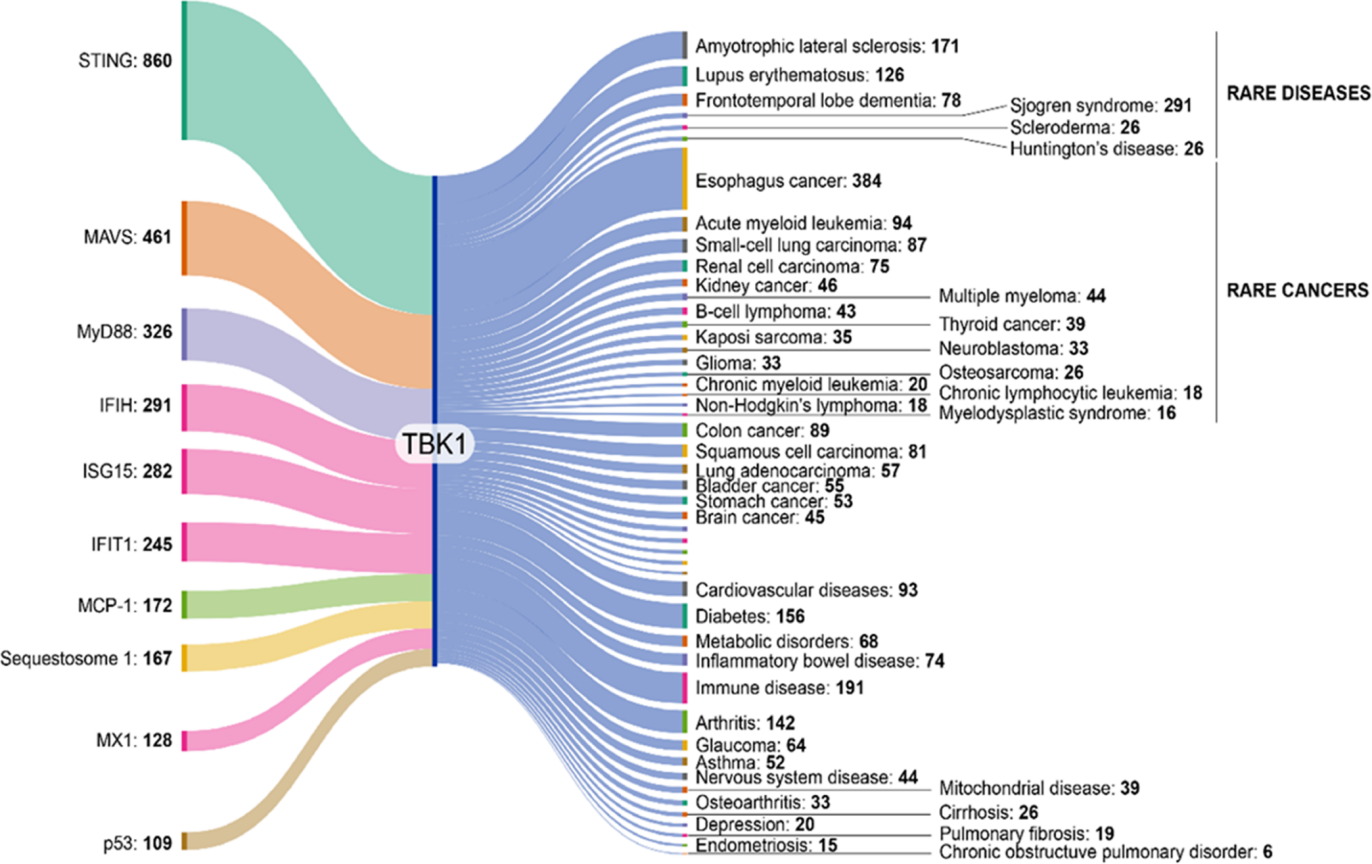
Sankey graph showing co-occurrences of TBK1 with other proteins (left column) and diseases (right column) based on CAS indexing. Data includes patent and journal publications from the CAS Content Collection for the period 2003–2023.

Using data from the CAS Content Collection, we determined co-occurrences of TBK1 with other protein targets as well as diseases (shown in the Sankey graph in Figure 3). Below we discuss briefly the role of TBK1 in diseases it co-occurs with in our data.

### Implication of TBK1 in Diseases

Due to its role in immune regulation, inflammation, autophagy, and cellular stress responses, dysregulation or dysfunction of TBK1 is implicated in various diseases.

- TBK1 has been associated with **autoimmune diseases** such as systemic lupus erythematosus (SLE), where dysregulated immune responses contribute to tissue damage and inflammation. TBK1 may play a role in the activation of immune cells and the production of inflammatory cytokines in autoimmune conditions.^22,31,50^ TBK1 is also responsible for regulating the immune response in multiple sclerosis, rheumatoid arthritis, inflammatory bowel disease, and psoriasis among others
- There is growing evidence linking TBK1 dysfunction to **neurodegenerative disorders** such as amyotrophic lateral sclerosis (ALS) and frontotemporal dementia (FTD). Mutations in the TBK1 gene have been identified in individuals with ALS-FTD spectrum disorders, suggesting a potential role for TBK1 in the pathogenesis of these diseases.^15,42^
- TBK1 is involved in the host immune response to **viral infections** by inducing the production of type I interferons and other antiviral proteins. Dysregulation of TBK1 signaling pathways may impact the ability of the immune system to control viral infections, leading to increased susceptibility to viral diseases.^51−53^
- TBK1 has been implicated in **cancer** development and progression in various ways. It can promote tumor growth, survival, and metastasis by enhancing cell proliferation, inhibiting apoptosis, and modulating the tumor microenvironment. Dysregulated TBK1 signaling has been observed in several types of cancer, including lung cancer, breast cancer, and melanoma.^34,54,55^ For instance, TBK1 has been shown to activate the NF-κB pathway, which is generally involved in inflammation, cell proliferation, and resistance to apoptosis in cells. This can eventually lead to tumor growth and proliferation. TBK1 phosphorylates STING, which in turn recruits IRF3 for phosphorylation by TBK1. Phosphorylated IRF3 dimerizes and then enters the nucleus, where it functions with NF-kB to turn on the expression of type I interferons and other immunomodulatory molecules. TBK1 activates several signaling pathways such as AKT-mTOR and MYC that are responsible for tumor cell survival. TBK1 also plays an important role in the IRF3 pathway that is important in generating an antiviral response, any disruption in these pathways is linked to tumorigenesis. In addition, TBK1 is also known to activate transcription factors responsible for epithelial-mesenchymal transition (EMT) which can lead to cancer metastasis. It can also influence the tumor microenvironment by modulating immune cell activity and causing cytokine release which can cause tumor progression. It is also implicated in resistance to different cancer therapies as it can inhibit apoptosis and promote cancer cell survival. TBK1 is also involved in crosstalk between other cancer-linked pathways in the cell such as KRAS, PI3K, and EGFR pathway. Due to all these factors any mutation in TBK1 gene can be linked to cancer.
- TBK1 may also play a role in metabolic regulation and energy homeostasis. Dysregulation of TBK1 activity has been linked to **metabolic disorders** such as obesity and insulin resistance, although the precise mechanisms remain to be fully elucidated.^38,39,56^

### Need of TBK1 Inhibitors

Because of its implication in a large assortment of diseases and disorders, TBK1 is a promising therapeutic target for the development of drugs aimed at modulating immune responses and treating various diseases.^57−59^ TBK1 inhibitors are compounds that can selectively block the activity of TBK1, potentially offering therapeutic benefits in various diseases where TBK1 is dysregulated or overactive. Research into TBK1 inhibitors has gained considerable attention due to the role of TBK1 in immunity, inflammation, and other cellular processes, as well as its implication in diseases such as cancer, autoimmune disorders, and neurodegenerative conditions.^14,15,34^ Several small molecule inhibitors targeting TBK1 have been developed and studied in preclinical and early clinical research. These inhibitors typically work by binding to specific regions of TBK1 and interfering with its kinase activity, thus preventing its downstream signaling and biological effects.^25,26,57,60,61^

One of the primary motivations for developing TBK1 inhibitors is their potential as anticancer agents. TBK1 has been implicated in promoting tumor growth and progression in certain types of cancer by enhancing cell survival, proliferation, and metastasis. Inhibiting TBK1 activity could potentially suppress these cancer-promoting effects and enhance the efficacy of other anticancer treatments.^29,34,58^

Furthermore, TBK1 inhibitors are also being investigated for their potential in treating autoimmune disorders and inflammatory conditions. Since TBK1 plays a role in the regulation of immune responses and inflammation, inhibiting its activity could help modulate aberrant immune activation and reduce inflammation associated with autoimmune diseases.^22,31,62^

Despite promising preclinical results, the development of TBK1 inhibitors faces several challenges, including achieving sufficient selectivity to minimize off-target effects, optimizing pharmacokinetic properties for effective delivery and distribution in the body, and ensuring safety and tolerability in clinical settings. Overall, while TBK1 inhibitors represent a promising avenue for therapeutic intervention in various diseases, further research and development efforts are needed to fully realize their clinical potential and address the challenges associated with their development.

### Why There Are No Successful TBK1 Inhibitors on the Market

Our search failed to identify any current US FDA-approved TBK1 inhibitors available on the market. The immunomodulatory drug Amlexanox which was approved by US FDA for the treatment of aphthous ulcers in 2004,^63^ was later discontinued in 2014 due to the associated undesired adverse reactions of the formulation.^64^ Amlexanox-loaded nanoliposome formulation are being currently developed as a potential alternative for the localized oromucosal delivery of the drug.^64^

The lack of US FDA-approved TBK1 inhibitors at present can be attributed to several factors:

(i) TBK1 is involved in multiple cellular processes, including immune regulation, inflammation, autophagy, and stress responses. The complexity of TBK1 signaling complicates the progress in discovery of TBK1 inhibitors. Developing inhibitors that selectively target TBK1’s pathological functions while sparing its essential physiological roles is challenging.
(ii) Achieving sufficient selectivity is crucial when developing kinase inhibitors to minimize off-target effects and potential toxicities. Designing compounds that specifically inhibit TBK1 without interfering with other kinases or cellular pathways can be difficult.
(iii) The process of drug development, from discovery to approval, is lengthy and resource intensive. Developing TBK1 inhibitors with desirable pharmacokinetic properties, efficacy, and safety profiles requires substantial investment in preclinical research, clinical trials, and regulatory approval processes.
(iv) Even if promising TBK1 inhibitors are identified in preclinical studies, their clinical efficacy and safety must be rigorously evaluated in human clinical trials. Negative results or unforeseen complications in clinical trials can delay or halt the development of candidate inhibitors.
(v) The prioritization of research and funding allocation in the pharmaceutical industry and academic institutions also influences the pace of drug development. While TBK1 inhibitors hold promise for therapeutic intervention in various diseases, competing priorities and resource constraints may impact the rate of progress in this area.

Despite these mitigating factors, a few key players that are involved in research related to TBK1 are shown in Figure 2 and include well-known companies such as Merck, Novartis, Gilead Sciences and Bayer. Other key players include the PROTAC-focused biotechnology company Arvinas,^65^ the Drug Discovery CRO Domainex,^66^ Asuragen^67^ which appears to be centered around molecular diagnostics in oncology and other fields as well as the China-based drug development company Shenzhen Chipscreen Biosciences.^68^ Examples of patents by these organizations mostly consist of exploring various scaffolds including pyrimidine- (WO2019079375;^69^ US8962609^70^) and heteroarylbenzimidazole-based (WO2017207534^71^) with the aim of developing small molecule inhibitors (WO2017106556^72^). Other examples include development of proteolysis targeting chimeras (PROTACs) against TBK1 by Arvinas (WO2016197114^73^).

### How IC50 Values Work for Inhibitors

The IC50 (half-maximal inhibitory concentration) value is a measure used in pharmacology and biochemistry to quantify the potency of an inhibitor, particularly in enzyme inhibition studies. It represents the concentration of an inhibitor required to inhibit 50% of the activity of a biological or biochemical target, such as an enzyme or a cellular process. It represents the most widely used and informative measure of a drug’s efficacy.^74,75^

In a typical experimental setup to determine the IC50 value of an inhibitor, varying concentrations of the inhibitor are tested against a fixed concentration of the target enzyme or biological process. The activity of the target is measured in the presence of each inhibitor concentration. The data obtained from the experiment is used to plot a dose–response curve, where the concentration of the inhibitor is plotted on the *x*-axis, and the remaining activity of the target (expressed as a percentage of the uninhibited activity) is plotted on the *y*-axis. As the concentration of the inhibitor increases, the activity of the target decreases. The IC50 value is determined by finding the concentration of the inhibitor that corresponds to 50% inhibition of the target activity on the dose–response curve. This concentration is the IC50 value. It represents the potency of the inhibitor—the lower the IC50 value, the more potent the inhibitor, as it achieves significant inhibition at lower concentrations. A low IC50 value indicates that the inhibitor is effective at lower concentrations, meaning it can achieve significant inhibition of the target with relatively low doses. Conversely, a high IC50 value indicates that higher concentrations of the inhibitor are needed to achieve the same level of inhibition, suggesting lower potency. IC50 values can be used to compare the potency of different inhibitors targeting the same biological target. The inhibitor with the lower IC50 value is generally considered more potent and may be more suitable for further development as a therapeutic agent or research tool.

In brief, the IC50 value is a quantitative measure of the potency of an inhibitor, representing the concentration required to inhibit 50% of the activity of a biological target. It is an essential parameter in drug discovery and enzyme inhibition studies, helping researchers evaluate and compare the effectiveness of different inhibitors.

## QSAR Modeling of TBK1 Inhibitors

### Computer Modeling of Kinase Inhibitors and Computer-Aided Drug Design

Computer modeling plays a significant role in the design of kinase inhibitors, which are crucial in drug discovery and development.^76,77^ In computer-aided drug design, predicting the IC50 values of potential drug candidates is crucial for assessing their potency in inhibiting the target enzyme or protein.

Quantitative structure–activity relationship (QSAR) models relate the chemical structure of compounds to their biological activity, including IC50 values. By analyzing a data set of known inhibitors with experimental IC50 values, QSAR models can be used to predict the IC50 values of new compounds. Molecular descriptors such as molecular weight, lipophilicity, hydrogen bonding capacity, and electronic properties are used to characterize the compounds and correlate them with their IC50 values.^78,79^

In structure-based drug design molecular docking simulations predict the binding mode and affinity of small molecules to the target protein’s active site.^80−82^ Compounds with favorable docking scores are more likely to have lower IC50 values. Molecular dynamics simulations can further refine the binding poses and assess the stability of the protein–ligand complex, providing insights into the dynamic behavior that may influence IC50 values. In ligand-based drug design pharmacophore modeling identifies the essential structural features required for binding to the target protein.^83,84^ Compounds that match the pharmacophore features are likely to exhibit activity, including potency measured by IC50 values. Similarity searching compares the chemical features of potential drug candidates to known active compounds with known IC50 values, enabling the prediction of potency based on structural similarity.

Machine learning algorithms, such as support vector machines (SVM), random forest, or neural networks, can be trained on large data sets of compounds and their corresponding IC50 values to predict the potency of new compounds. Deep learning approaches, including convolutional neural networks (CNN) and recurrent neural networks (RNN), can capture complex relationships between chemical structures and biological activity, improving the accuracy of IC50 predictions.^85−88^

### QSAR Modeling of TBK1 Inhibitors Based on Available IC50 Experimental Data

**Figure 4:**
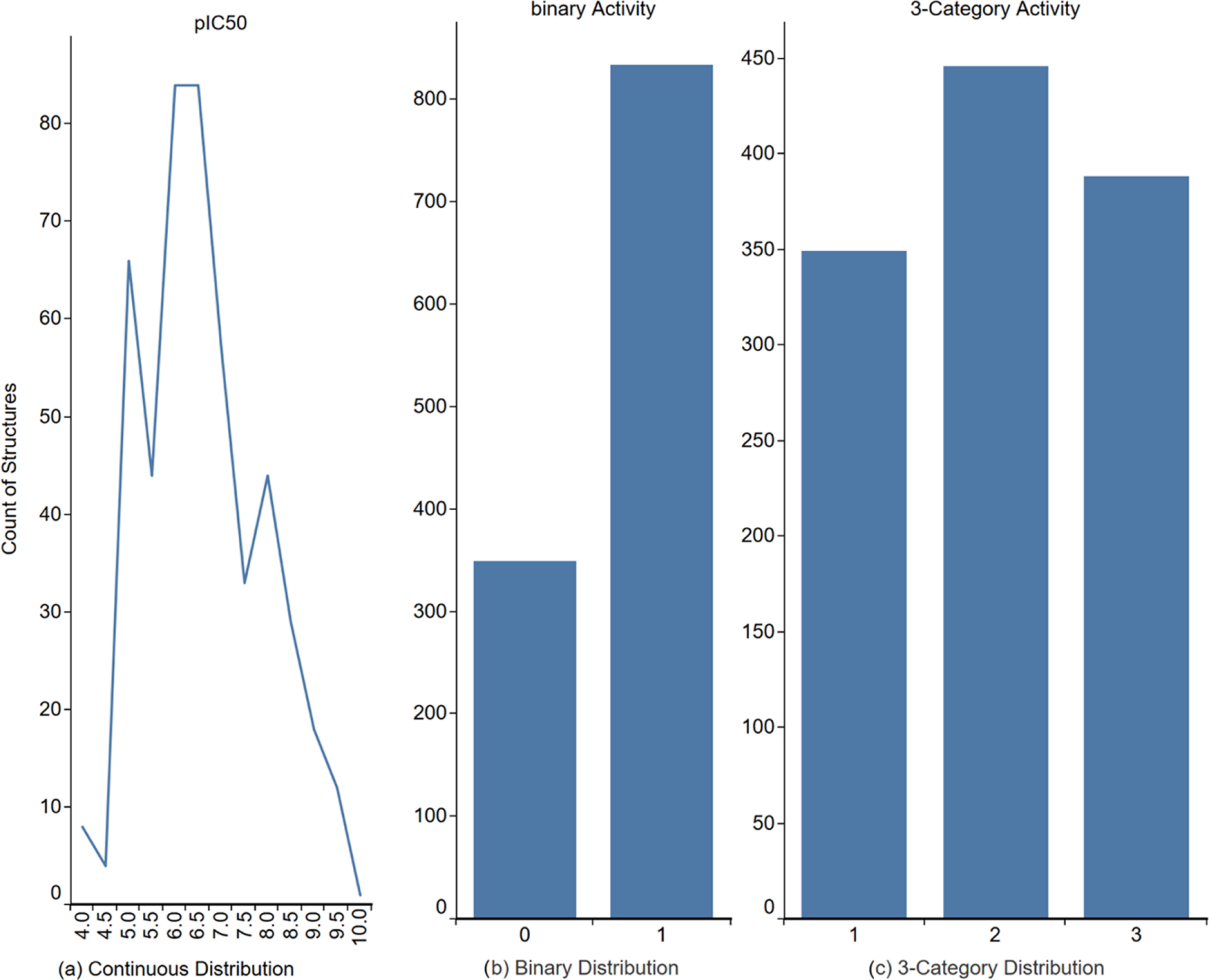
Distributions of pIC50 of the training set data for the three predictive QSAR models developed – (A) regression model, (B) binary model and (C) 3-category distribution.

Our main goals in this study were to develop the best possible predictive models for TBK1 inhibitors that could be used by research scientists in their quest to discover effective drugs against wide range of diseases such as cancer, viral infections, and inflammatory disorders. We also made a conscious effort to explain why the models work based on the top molecular descriptors that appear in the models which may provide an invaluable insight into the mechanism of the TBK1 inhibition. To accomplish this, we used the CAS Content Collection to extract all available data associated with target protein TBK1 including IC50 and pIC50 values of inhibitors. After removing all duplicate structures, records without structural information, salts, and mixtures we arrived at our final training set with 1,183 compounds, all single organic chemicals. Upon close examination of the data, the IC50 values of more than half of the structures have been entered as active (IC50 < 0.1 μM or IC50 < 0.001 μM) or as inactive (IC50 > 10 μM) (Figure S1). The data distribution of pIC50 of the training set is outlined in Figure 4. These specifics of the data entry prompted us to pursue 3 distinctive types of predictive models. For the continuous distribution, (a) regression models, for the binary distribution, (b) binary models, and for the 3-category distribution, and (c) multiclassification models (Figure 4).

As already mentioned above, only a fraction of the data is presented with their exactly measured concentration. For the development of the regression models, we only used the 475 structures with the exact experimental measurement of IC50 and omit any data entered as active/inactive as they introduce a significant penalty for the accuracy of the regression models. For the classification models we used the entire set of 1,183 chemicals with 349/834 distribution of active/inactive and a breakpoint at pIC50 = 7 for the binary models and 349/446/388 of active/marginal/inactive and breakpoints at pIC50 = 7 and 8 for the 3-category models.

We explored several different sets of molecular descriptors, as it is not known beforehand what features best correlate with the inhibition of TBK1. In this investigation we used our proprietary CAS fingerprint,^89^ the fragments generated by the Fragment–Functional Analysis,^90^ and the available molecular descriptors in RdKit.^91^ A brief summary of all molecular descriptors is given in Table 1.

**Table 1:** Molecular Descriptors Used in the Study

**molecular descriptors**	**short summary**
CAS fingerprint^89^	CAS proprietary fingerprint consists of over 7,000 molecular features
CAS fragment–functionality^90^	CAS proprietary fragment–functionality analysis
Morgan fingerprint^92^	RdKit - the hashed Morgan fingerprint for a molecule (radius = 3; length = 2048)
MACCS keys^93^	RdKit -166 public MACCS keys
Atom Pairs^94^	RdKit - the atom-pair fingerprint for a molecule
Topological Torsion^95^ fingerprints	RdKit - the hashed topological-torsion fingerprint for a molecule
Crippen LogP, and MR^96^	RdKit - the Wildman-Crippen logp, mr
MQNs^97^	RdKit - the Molecular Quantum Numbers
PEOE_VSA, SMR_VSA, SlogP_VSA^98^	RdKit - atoms van der Waals surface area (VSA) descriptors
BCUT2D^99^	RdKit - diagonal elements: atomic mass, Gasteiger charge, Crippen logP, Crippen MR
FractionCSP3^91^	RdKit - the fraction of C atoms that are SP3 hybridized
Topological descriptors^91^	RdKit - various topological descriptors

An automated machine learning platform, DataRobot (https://www.datarobot.com/), was used to train and evaluate performance of more than 70 different machine learning algorithms. DataRobot is also employed to build informative features selected from molecular descriptors. To counter the overfitting, a well-known problem in machine learning, and to estimate the statistical performance of the models, a common 5-fold cross-validation procedure has been utilized for all models in this study.

The model building procedures is as follow: first the initial set of structures is split into two subsets with 80% of the structures utilized as model building set and the remainder 20% utilized as a holdout test set. The holdout test set is kept aside and is not used in the development of QSAR models. Instead, it is utilized as control set to assess the accuracy of the models as the chemicals in the holdout set are external with respect to the model building procedure. The model building set is subject to a 5-fold cross-validation procedure for internal validations. In each of the five iterations of this approach, 80% of the model building set is used to build a model, and 20% is held as a test set. Across the entire process, then, every record is held out as validation in one part of the process, yet all records are made available to the model. Grid search was used as the default method for hyperparameter optimization.

As already mentioned, we pursued 3 types of models to predict the TBK1 inhibition: binary classifiers, three category classifiers, and regression models. Number of common matrices were used to evaluate the statistical performance of the predictors.

#### Binary Classifiers

Area Under the ROC Curve (AUC) – measures the ability to distinguish ones from zeros.

Sensitivity/Recall–measures the probability of a positive test result.
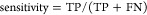
1

Specificity–measures the probability of a negative test result.
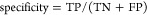
2

Precision - fraction of relevant instances among the retrieved instances.
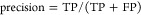
3

Accuracy–fraction of the correctly classified samples.
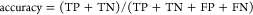
4

F1_score–measures the predictive skill of a model by elaborating on its class-wise. It combines two competing metrics- precision and recall scores of a model.
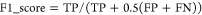
5

where: TP - true positives, TN - true negatives, FP - false positives, FN - false negatives.

#### Three Category Classifiers

Logarithmic loss–measures the inaccuracy of the predicted probabilities.
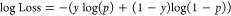
6where: *y*-actual output, *p*-probability predicted by the logistic regression.

Accuracy–fraction of the correctly classified samples (eq 4) for each class.

Balanced accuracy–average of sensitivity (eq 1) per target class.

Area Under the ROC Curve (AUC)–measures the ability to distinguish ones from zeros.

#### Regression Models

*R*^2^ – measures the proportion of total variation of outcomes explained by the model
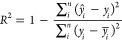
7

Root mean square error (RMSE) – measures the inaccuracy of the predicted mean values.
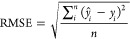
8

Mean absolute error (MAE) - measures the inaccuracy of predicted median values.
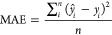
9where: *ŷ*_*i*_ is a predicted value, *y*_*i*_ is a real value, *y̅*_*i*_ is a mean value, over all samples, and *n* is the number of samples.

In addition to the common internal and external QSAR validations, the models were also evaluated by making predictions against a completely independent external validation set of 21 TBK1 inhibitors with experimental IC50 data, found in recent publications^26,100^ that are not part of the initial data set of 1183 structures.

## Results and Discussion

To derive the best predictive models, we first computed all molecular descriptors. For CAS fingerprint^89^ and Fragment-functional analysis^90^ we employed our own proprietary software. For Fragment-functional analysis^90^ over 66,000 single fragments were generated after splitting the chemical structures of the training set into subfragments. The RdKit^91^ molecular descriptors were computed utilizing the python implementation of RdKit and the SMILES^101^ strings of the chemicals in the training set. As scientists and regulatory agencies around the world may have diverse needs for how the predictions are presented as well as the particulars of the input data (as explained in section “Data distribution” above) we probed three distinct types of predictive models: Regression, Binary, and 3-Category models.

**Figure 5:**
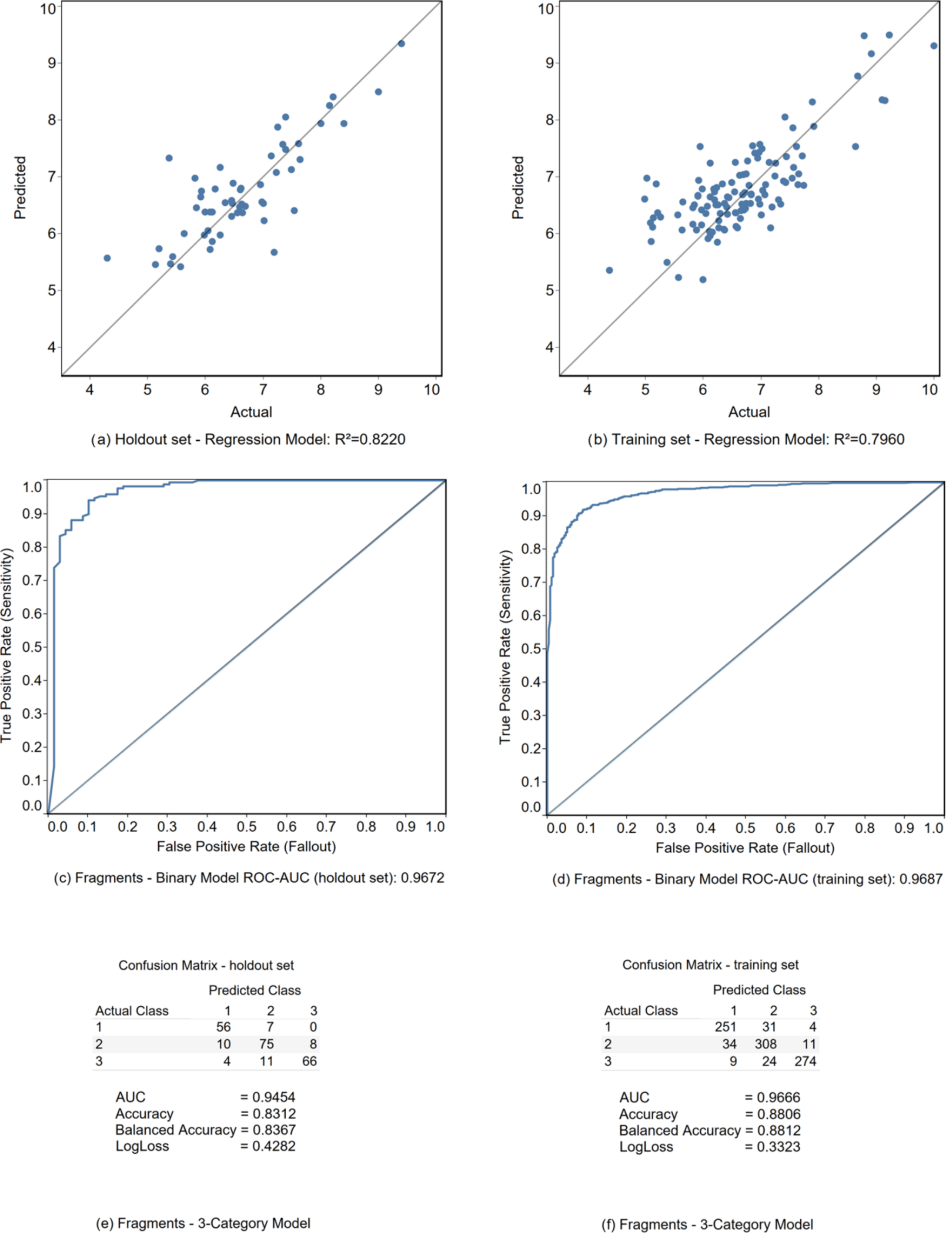
Statistical performance of the models derived by the Fragments-functional descriptors.

Considering both the performance of the common statistical matrices and the predictions of the holdout test set and the independent external set, the best overall predictive models were derived from the fragment-functional analysis descriptors.^90^ Graphical representation of the statistical performance of these models is outlined in Figure 5.

To assess the predictive abilities of our models we used internal and external validation tests.^102−104^ For the regression model we obtained *R^2^*_holdout_ = 0.822 (Figure 5a) and *R^2^*_cros-validation_ = 0.796 (Figure 5b) for the external and internal validations, respectively. The difference *R^2^*_holdout_ – *R^2^*_cros-validation_ of 0.026 clearly indicates that there are low overfitting ramifications with the regression model. The same holds true for the classification models, where we consider AUC_holdout_ and AUC_cros-validation_. We achieved AUC differences of 0.0015 and 0.0212 for the binary (Figure 5c,d) and 3-category (Figure 5e,f) models, respectively.

We also utilized Tropsha’s statistical characteristics^102^ to assess the external predictability of the regression model. We computed (as described in ref (102)) *k*, *k*′, *R*_0_^2^, and *R*′_0_^2^, the slopes and correlation coefficients between predicted vs observed (and vice versa: observed vs predicted) activities of the structures in the holdout test set.

A QSAR model is acceptable if the following conditions are met:^102^

1. *R^2^*_cross-validation_ > 0.5
2. *R^2^*_test set_ > 0.6
3. |(*R^2^*_test set_ – *R*_0_^2^)/*R^2^*_test set_ | < 0.1 or |(*R^2^*_test set_ – *R*′_0_^2^)/*R^2^*_test set_| < 0.1
4. 0.85 ≤ *k* ≤ 1.15 or 0.85 ≤ *k*′ ≤ 1.15

For the regression model we have

*R^2^*_cross-validation_ = 0.796 (which is >0.5)

*R^2^*_holdout_ = 0.822 (which is >0.6)

*R*_0_^2^ = 0.8879 and *R*′_0_^2^ = 0.9518 → | (0.822–0.8879)/0.822 | = 0.08 (which is <0.1)

*k* = 1.0547 and *k*′ = 0.9502 - both are in the range of 0.85–1.15.

Thus, satisfying all the above conditions.

Y-scrambling validation is a widely used technique^105−107^ to evaluate the robustness of QSAR models and to ensure that the developed models are not derived due to chance. In this test, the dependent variable (observed activity) randomly shuffles while keeping the independent variables (molecular descriptors) unchanged, and a new model is derived. This process is repeated several times and the values of *R^2^*_test,_ and *R^2^*_cross-validation_ are recorded. The values of *R^2^*_test_ and *R^2^*_cross-validation_ are expected to be low, ensuring the developed QSAR is robust and not derived due to chance. For the current study we run 10 Y-scrambling tests, and the results are presented on Table 2. All values of *R*^2^_test_ and *R^2^*_cross-validation_ are below 0.1 thus confirming that the regression model is not derived due to chance.

**Table 2:** Y-scrambling Test Results

**#**	***R^2^***_**cross-validation**_	***R***^**2**^_**test**_
1	0.0205	0.00084
2	0.0119	0.00877
3	0.0072	0.00356
4	0.0187	0.00442
5	0.0013	0.00919
6	0.0101	0.0118
7	0.0200	0.0347
8	0.0200	0.00222
9	0.0227	0.00625
10	0.0225	0.00062

The results for the predictions of the external test chemicals are presented in Table 3. The results obtained from all molecular descriptor sets and models in this investigation are available in the Supporting Information material (Figures S4–S24, and Tables 2–4).

**Table 3:** Predictions for the Independent External Set of 21 Compounds

**#**	**CAS RN**	**pIC50-observed**	**binary-observed breakpoint at pIC50 = 7**	**3-category-observed**^a^	**pIC50-predicted**	**binary-predicted**	**3-category-predicted**^a^
1	68301-99-5	4.26	0	1	5.36	0	1
2	2116443-03-7	4.62	0	1	4.67	0	1
3	2116445-80-6	5.54	0	1	6.22	0	1
4	2116445-76-0	4.00	0	1	5.67	0	1
5	2116445-77-1	4.44	0	1	5.34	0	1
6	2116445-78-2	4.14	0	1	5.33	0	1
7	unknown^b^	5.35	0	1	5.67	0	1
8	2116445-85-1	4.48	0	1	4.73	0	1
9	70529-18-9	6.40	0	1	5.89	0	1
10	2116445-81-7	5.54	0	1	5.54	0	1
11	2116445-82-8	4.00	0	1	5.80	0	1
12	1056634-68-4	7.24	1	2	7.16	1	3
13	2116443-62-8	6.70	0	1	5.75	0	1
14	2243281-75-4	8.05	1	3	7.80	1	2
15	2243281-77-6	7.70	1	2	7.80	1	2
16	2322365-47-7	8.30	1	3	8.07	0	3
17	81267-65-4	6.02	0	1	5.92	0	1
18	1835675-67-6	8.55	1	3	8.45	0	1
19	2101906-58-3	7.86	1	2	7.76^c^	1	2
20	1903773-70-5	7.70	1	2	6.61^c^	0	1
21	2020003-22-7	6.89	0	1	5.21	0	1
					*R*^2^ = 0.69	accuracy = 0.86	accuracy = 0.81

a Category 1 (pIC50 < 7); Category 2 (pIC50 < 8); Category 3 (pIC50 ≥ 8).

b SMILES: CC(C)C1C=CC(=C2C=1)OC3N=C(N)C(C(=O)OCCN(C)C)=CC=3C2=O.

c Warning: The prediction might be incorrect as the chemical lies outside of the applicability domain.

**Table 4:**
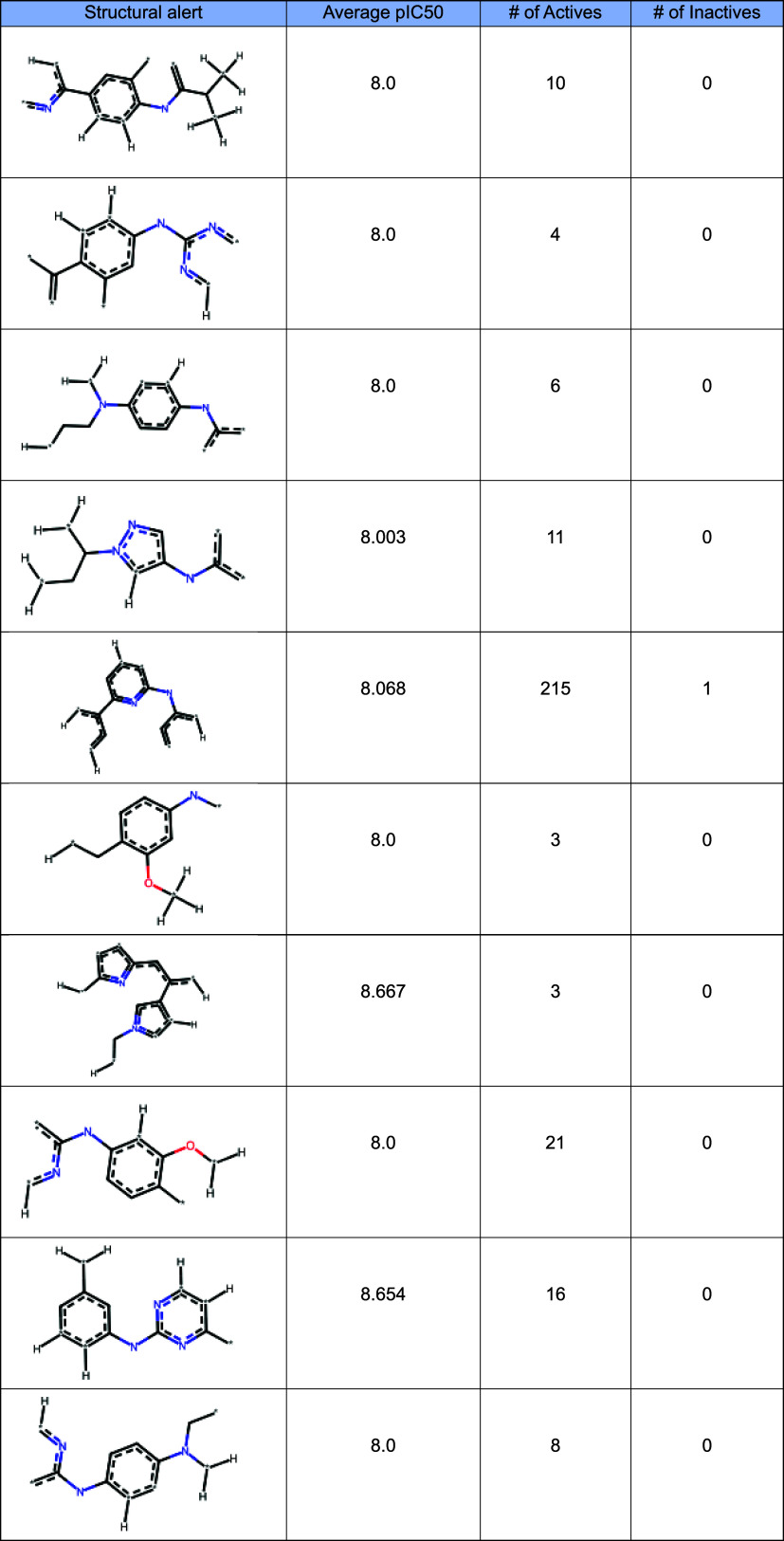
Structural Alerts for the Fragment Binary Model

A quick glance at the results in Table 3 reveals that while the statistical criteria used to validate the models meet or exceed the common standards for good predictors, *R*^2^ of 0.69 for the predictions of the independent external set with the regression model, although acceptable, is noticeably lower than the *R*^2^ of the holdout test set (0.822). The lower value of *R*^2^ for the external set can be attributed to the fact that these structures are external in the true sense of the word. While the chemicals in the holdout test set are external for the model building procedure, they are not entirely external for the model building data with respect to structural similarity. Most of the chemicals in the initial training set originate from different studies; however, a single study often contains series of chemical compounds that may bear some structural similarities. In contrast, the TBK1 inhibitors in our independent external test set are not part of the initial set and any similarities that might exist can be attributed to chance. With much more chemical diversity for the classification models where the initial set is 1,183 chemicals strong, both the binary and 3-category predictors perform well in the validation of the holdout set as well as the predictions of the independent external set.

One particularly important aspect of QSAR modeling is defining the applicability domain^103,105−109^ of the predictors. Or in other words to determine the chemical space where the models are suitable to make quality predictions and avoid a potential misuse of the results. Thus, the predictions for new molecules obtained from a QSAR model are acceptable only if the new molecules fall inside the applicability domain of that model. The applicability domain is the chemical space defined by all molecular descriptors used to build the QSAR model. For a fragment-based methodology the molecular descriptors are the structural alerts obtained by splitting the chemical structures of the training set into all possible subfragments. In the current study our algorithm generated over 66,000 unique fragments from the compounds in the training set which define the domain of applicability for our TBK1 predictive models.

There are several ways to graphically illustrate the applicability domain of QSAR models. In the current study we utilized the Williams plot which is commonly used^105−109^ and represents the applicability domain as a plot of Leverage (*h*) vs Standardized Residuals (σ).

Leverage of a given chemical structure *h_i_* is defined as
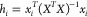
10where: *x*_*i*_ is the descriptor vector of the *i*th structure; *X* is the descriptor matrix of the training set used to build the model

The warning leverage h* is defined as
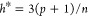
11where: *p* is the number of descriptors in the model; *n* is the number of chemicals in the training set

Test chemicals with *x*_*i*_ < h* are considered reliably predicted.

**Figure 6:**
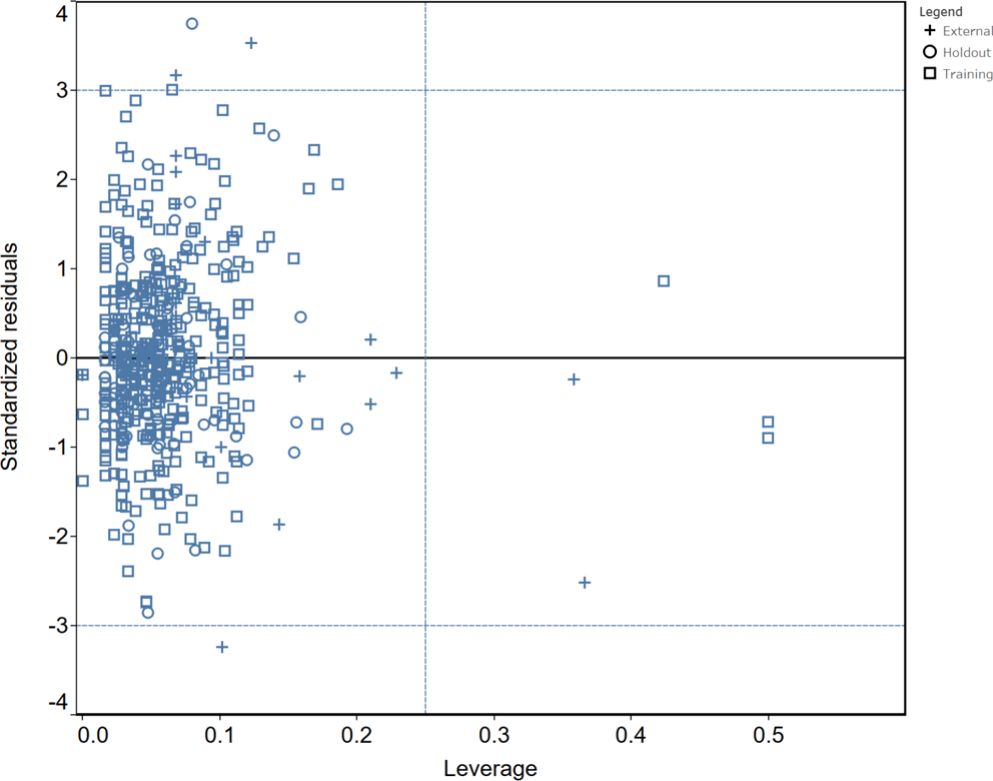
Williams plot of the applicability domain.

On Figure 6. is shown the Williams plot for the regression model, where the applicability domain is defined within ±3σ and a leverage threshold h* = 0.25. From the plot, it is apparent that all compounds of the training set are within the applicability domain except 3 with leverage values greater than the warning h*. These 3 chemicals could influence the performance of the model, however, like^106^ their standard residuals are well within the established limits and thus, not model outliers to be considered for removal from the training set. There are also 2 structures from the external test set with *h* > h* marked (see footnote c) in Table 3 as warning predictions. There are also 3 external set compounds outside of the established limits of the standard residuals, which explains the lower *R*^2^ of the independent external set.

In our study we did not try to define upfront what structural alerts are statistically significant/important. Instead, we let the machine learning algorithms decide the importance of the molecular descriptors. In Table 4. are listed some of the most significant structural alerts. In fragment-based drug design, structural alerts serve as critical tools for identifying promising fragment scaffolds with potential to bind to a target protein. By recognizing specific molecular features associated with desired or undesired properties, these alerts enable early prediction of a fragment’s suitability for progression into lead optimization. This knowledge helps focus drug discovery efforts on compounds with higher likelihood of success while minimizing the risk of developing molecules with liabilities such as toxicity or poor pharmacokinetics.

As has been described briefly in the introduction, TBK1 is composed of four domains–kinase domain, ubiquitin-like domain, scaffold/dimerization domain and TANK-binding domain. The structure of TBK1 has been determined with several structures from across different methodologies (X-ray, cryo-EM) and species and are available in the Protein Data Bank (PDB). Many of the reported structures of TBK1 are in the presence of an inhibitor such as the withdrawn drug amlexanox (CAS RN: 68302-57-8)^100^ and a highly selective small molecule inhibitor in development, BAY-985 (CAS RN: 2409479-29-2).^110^ Both inhibitors appear to be competitive in nature, binding around the same area as ATP would.

A few key features emerge:

1. The binding site itself appears to consist of a mixture of charged residues and polar residues (Glu87, Arg25, Thr156, Cys89) as well as hydrophobic residues (Leu15, Val23, Ala33, Gly92).
2. Ability to H bond with Cys89 appears to be important.
3. Besides this, H bond interactions with other residues such as Glu87 and Thr156 also appear to be crucial for the inhibitory effect.
4. There also appears to be a size limit to the binding site wherein addition of bulk beyond a certain point is not tolerated and leads to a sharp decline in inhibitory activity.
5. Pushing the gatekeeper residue, Met 86, resulted in increased potency.

Based on the available information, it appears that the ability to H bond may be crucial for inhibitory effect at TBK1. Consequently, structural features capable of H bonding (donors and acceptors) are likely important. Also, inhibitors having variations of the purine moiety are likely to be effective since inhibitors have to compete with ATP for the binding site. Structural elements capable of engaging in van der Waals interactions with hydrophobic residues likely help boost inhibitory potency. Finally, structural elements that aid in pushing the gatekeeper residue Met 86, such as bulky substituents (5 or 6 membered rings) are likely to also increase inhibitory potency.

## Conclusions

TBK1 is a serine/threonine kinase involved in various signaling pathways, particularly those regulating immune responses, which pinpoints it as an important player in innate immunity, particularly in the regulation of type I interferon responses. Understanding its key molecular fragments and their interactions with receptors can help in the development of inhibitors or modulators for therapeutic purposes. In the last two decades, there has been a steady increase in interest in TBK1 from the scientific community, especially evident after 2019. Despite this continued and sustained interest and TBK1’s obvious involvement in a wide variety of diseases, targeting TBK1 effectively has been challenging. Notwithstanding the challenges, the pursuit of a TBK1 selective inhibitor is of interest to the scientific community and pharmaceutical entities.

In this study we developed a few machine learning models capable of predicting IC50 of small molecules inhibiting TBK1, a promising therapeutic target for the development of drugs modulating immune responses and treating wide range of diseases. We used the CAS Content Collection to assemble a training set of 1,183 chemical structures with experimentally measured IC50 toward TBK1. We explored several machine learning techniques combined with various molecular descriptors to derive and select the best TBK1 inhibitor QSAR models. Several structural alerts responsible for the mechanism of inhibition of TBK1 are also outlined and discussed. In the context of fragment-based drug design, such structural alerts can help identify promising fragments and predict potential liabilities, in order to guide lead optimization in drug discovery.

## Data Availability

Publications of TBK1 inhibitors were identified by optimizing a search of relevant terms on the CAS Content Collection using CAS STN. While the full data set is considered proprietary by CAS, the search string used for retrieval is included in the Supporting Information. TBK1 chemical inhibitors, the associated structural activity relationship data were extracted from these publications in CAS Content Collection.

## Supporting Information Available

Data modeling description (PDF)
