# Supplementary material for: In Silico Insights: QSAR Modeling of TBK1 Kinase Inhibitors for Enhanced Drug Discovery
Source: J Chem Inf Model. 2024 Sep 17;64(19):7488–502. doi: 10.1021/acs.jcim.4c00864 (PMC11480986; doi:10.1021/acs.jcim.4c00864)
Supplement: Supplementary file 1 — ci4c00864_si_001.pdf [file ci4c00864_si_001.pdf]

Supplementary information for:

In silico insights: QSAR modeling of TBK1 kinase inhibitors for enhanced drug discovery

Julian M. Ivanov,[1] Rumiana Tenchov,[1] Krittika Ralhan,[2] Kavita A. Iyer,[2] Shivangi Agarwal,[2] and Qiongqiong Angela Zhou[1]*

[1]CAS, A Division of the American Chemical Society, Columbus, Ohio 43210, United States

[2]ACS International India Pvt. Ltd., Pune 411044, India

*Corresponding author, email: qzhou@cas.org

Search query for identifying relevant documents

s (TBK1 or "TBK(w)1" or "TBK-1" or "TANK-binding kinase 1" or "Tank-binding kinase 1" or "TANK binding kinase 1" or "tank binding kinase 1" or "Serine/threonine-protein kinase TBK1" or "serine (w)threonine(w)protein kinase TBK1" or "NF-Kappa-B-Activating Kinase")

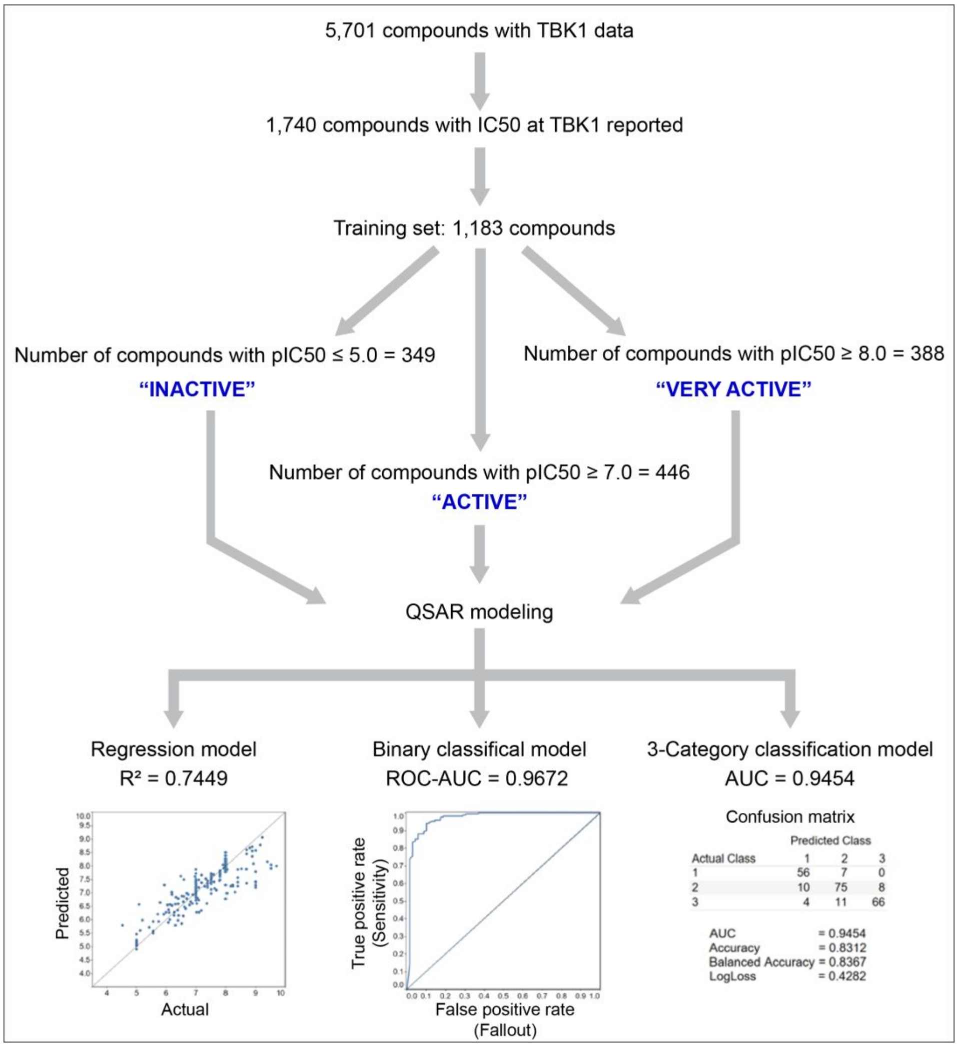

Figure S1. Schematic detailing steps involved in QSAR modeling of TBK1 inhibitors.

DataRobot model development procedure

Model development workflow:

The model development was done by performing a parallel heuristic search for the best model or ensemble of models, based on both the characteristics of the data and the prediction target. Several independent challenger models were developed by using the fundamental workflow involving multiple steps (Figure S2) as described below.

a) Rapid Data Ingestion: A modeling dataset is ingested that includes the prediction target.

b) Target Selection: DataRobot selects and builds regression blueprints when the selected target is continuous.

c) Automated Data Preparation: The input data is analyzed and advanced preprocessing steps are performed where the input data is partitioned into learning, validation, and holdout datasets.

d) Blueprints and ranking: DataRobot uses information about the selected target variable and predictors to define a set of candidate blueprints for analysis. The model is then trained and ranked based on an out-of-sample validation accuracy score.

e) Transparent Model Evaluation and Selection: The model accuracy is reviewed and evaluated by using metrics such as AUC, Log-Loss, and RMSE to select the best model.

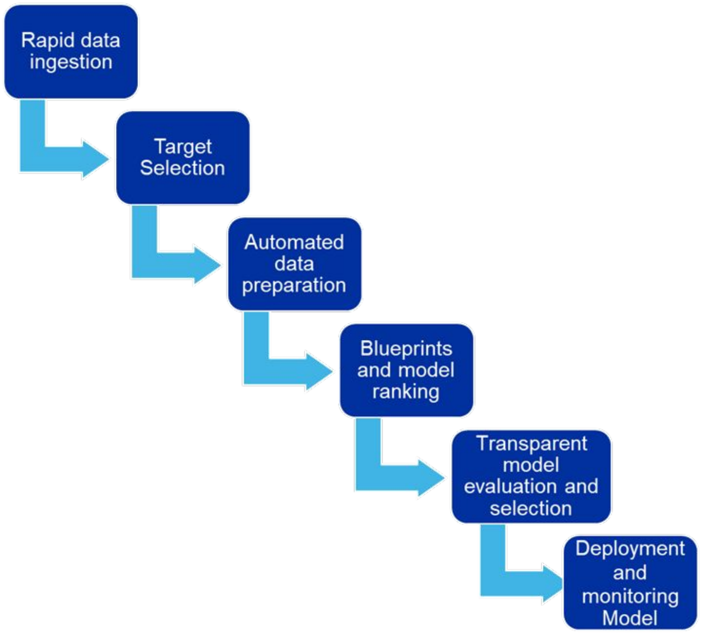

Figure S2: Flowchart demonstrating the workflow for the regression model

The modeling workflow consists of several elements that represent the end-to-end procedure for model fitting (figure S3).

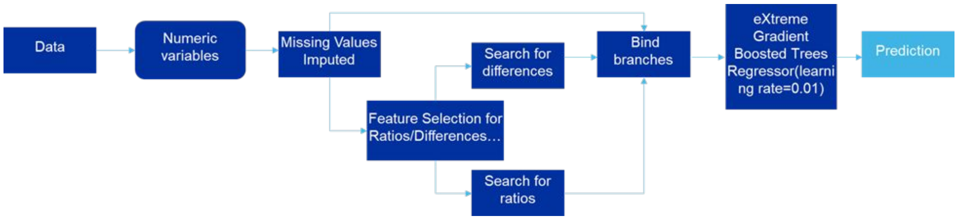

Figure S3: The elements of model fitting.

Cross-Validation procedure:

The best model was selected from among the various models generated. The stability of the model was judged by 5-fold cross-validation (CV). During CV process, 80% of data is available for training which is divided into 5 folds for cross-validation, and the rest acted as a holdout sample which is used for external validation. Figure S4 summarizes the CV process, where blue denotes

the training data and red denotes the holdout sample. The cross-validation RMSE was computed for each of the folds (table 1) which indicates how closely the model predicts the measured values.

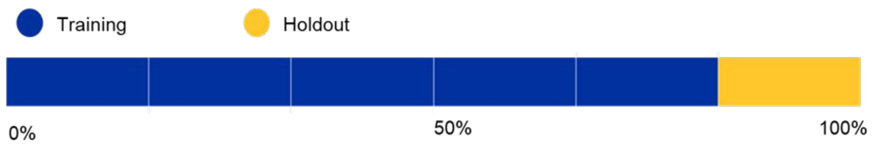

Figure S4. Cross-validation process during model building

Supplementary table 1. RMSE values for 5-folds cross-validation

Fold	Cross Validation Score (RMSE)
Fold 1	0.55324
Fold 2	0.47997
Fold 3	0.48887
Fold 4	0.44319
Fold 5	0.45149

Statistical measures

Model evaluation criteria: In this investigation we derived 3 different types of models to predict the TBK1 inhibition: binary classifiers, three category classifiers, and regression models. To estimate the statistical performance of the models, a few common matrices were used to evaluate the statistical performance of the predictors.

Binary classifiers:

Area Under the ROC Curve (AUC) – measures the ability to distinguish ones from zeros.

Sensitivity/Recall – measures the probability of a positive test result.

Sensitivity = TP / (TP + FN) (f1)

Specificity – measures the probability of a negative test result.

Specificity = TP / (TN + FP) (f2)

Precision - fraction of relevant instances among the retrieved instances.

Precision = TP / (TP + FP) (f3)

Accuracy – fraction of the correctly classified samples.

Accuracy = (TP + TN) / (TP + TN + FP + FN) (f4)

F1 – measures the predictive skill of a model by elaborating on its class-wise. It combines two competing metrics- precision and recall scores of a model.

F1 = TP / (TP + 0.5(FP + FN)) (f5)

where: TP - true positives, TN - true negatives, FP – false positives, FN - false negatives.

Three category classifiers:

Logarithmic loss – measures the inaccuracy of the predicted probabilities.

$$LogLoss = -(y\log(p) + (1-y)\log(1-p)) \quad \text{(f6)}$$

where: y-Actual output, p-probability predicted by the logistic regression.

Accuracy – fraction of the correctly classified samples (f4) for each class.

Balanced Accuracy – average of Sensitivity (f1) per target class.

Area Under the ROC Curve (AUC) – measures the ability to distinguish ones from zeros.

Regression models:

R Squared - measures the proportion of total variation of outcomes explained by the model

$$R^2 = 1 - \frac{\sum_i^n(\hat{y}_i - y_i)^2}{\sum_i^n(y_i - \bar{y}_i)^2} \quad \text{(F7)}$$

Root Mean Square Error (RMSE) – measures the inaccuracy of the predicted mean values.

$$RMSE = \sqrt{\frac{\sum_i^n(\hat{y}_i - y_i)^2}{n}} \quad \text{(f8)}$$

Mean Absolute Error (MAE) - measures the inaccuracy of predicted median values.

$$MAE = \frac{\sum_i^n(\hat{y}_i - y_i)^2}{n} \quad \text{(f9)}$$

where: $\hat{y}_i$ is a predicted value, y_i is a real value, $\overline{y}_i$ is a mean value, over all samples, and n is the number of samples.

1. TBK1 data modeling

The three types of models were developed using DataRobot v8.0.10. a) regression model b) binary classification model c) 3-category classification model. The 3-category model and binary model were built using the eXtreme Gradient Boosted Trees Classifier. The regression model was built by eXtreme Gradient Boosted Trees Regressor (learning rate =0.01).

1.1. Regression models

The Predicted vs Actual plot and the corresponding R^2 show how close the selected model is to the perfect predictor.

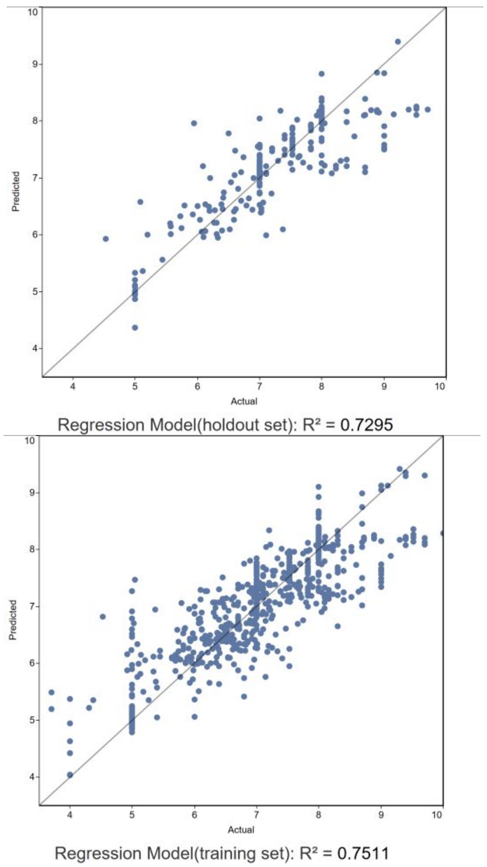

Figure S5. Predicted vs Actual plots for the regression model with CAS fingerprint.

The top descriptors that contributed to the biological activity are shown in Figure S6 Features impact. Feature Impact measures how important a feature is in the context of a model. It shows which features are driving model decisions the most. Feature Impact also helps improve the model by identifying unimportant or redundant features that can be dropped to improve model performance.

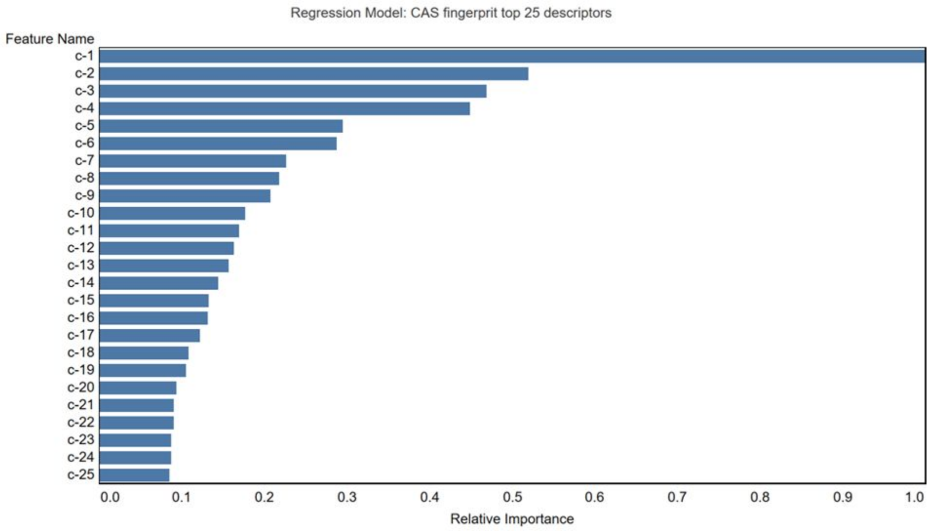

Figure S6. Features impact: top 25 features for the regression model with CAS fingerprint

Structures of the top 25 features for the regression model with CAS fingerprint are shown on Figure S7.

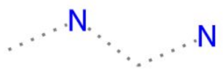

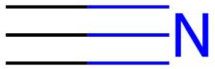

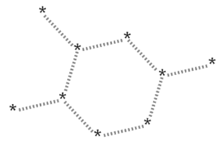

c-1 c-2 c-3

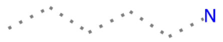

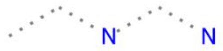

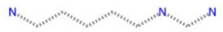

c-4 c-5 c-6

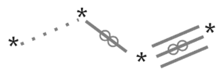

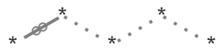

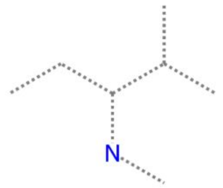

c-7 c-8 c-9

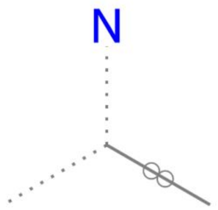

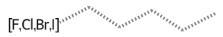

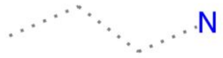

c-10 c-11 c-12

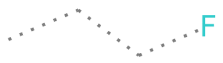

c-13

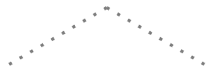

c-14

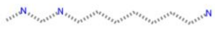

c-15

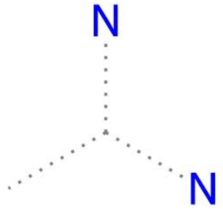

c-16

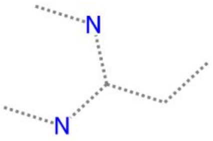

c-17

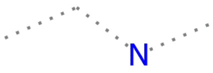

c-18

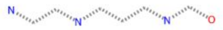

c-19

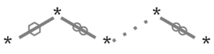

c-20

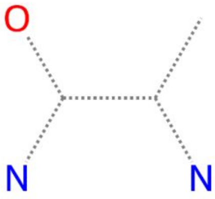

c-21

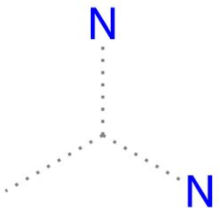

c-22

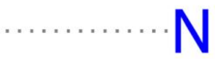

c-23

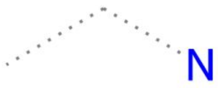

c-24

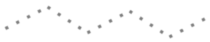

c-25

Figure S7. Structures of the top 25 features for the regression model with CAS fingerprint

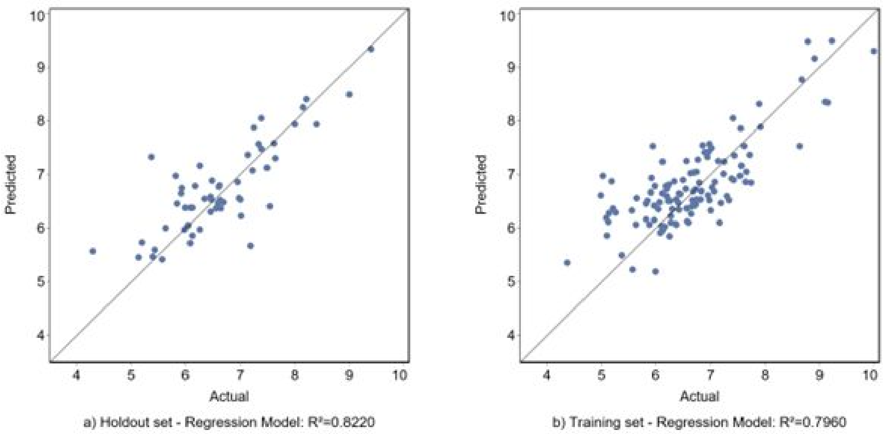

Figure S8. Predicted vs Actual plots for the regression model with CAS Fragment-functionality.

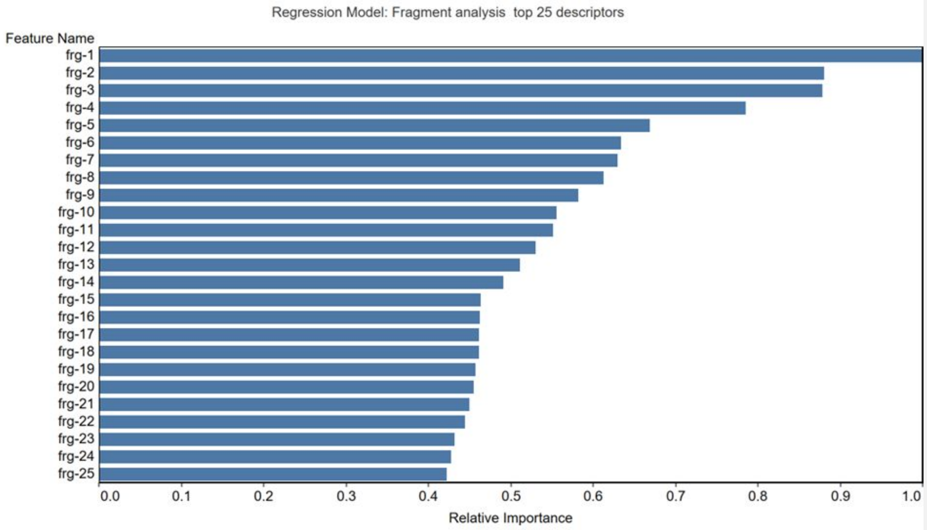

Figure S9. Features impact: top 25 features for the regression model with CAS Fragment-functionality

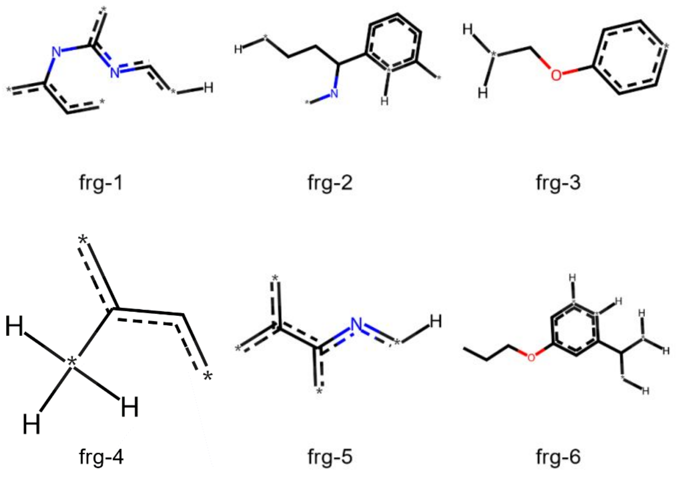

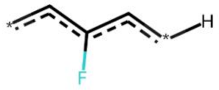

frg-7

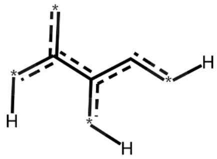

frg-8

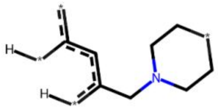

frg-9

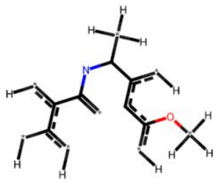

frg-10

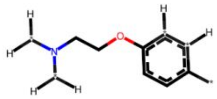

frg-11

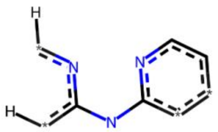

frg-12

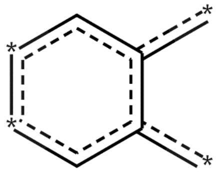

frg-13

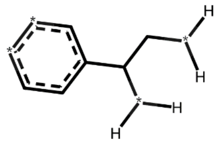

frg-14

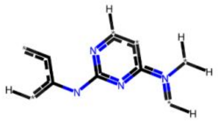

frg-15

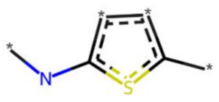

frg-16

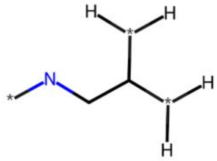

frg-17

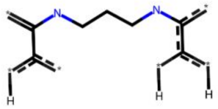

frg-18

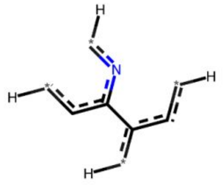

frg-19

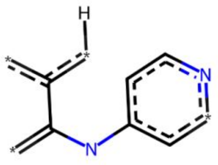

frg-20

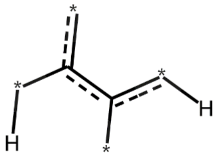

frg-21

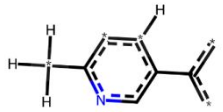

frg-22

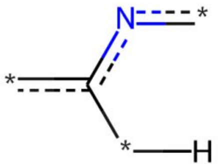

frg-23

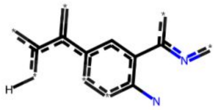

frg-24

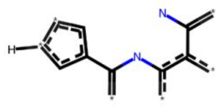

frg-25

Figure S10. Structures of the top 25 features for the regression model with CAS Fragment-functionality

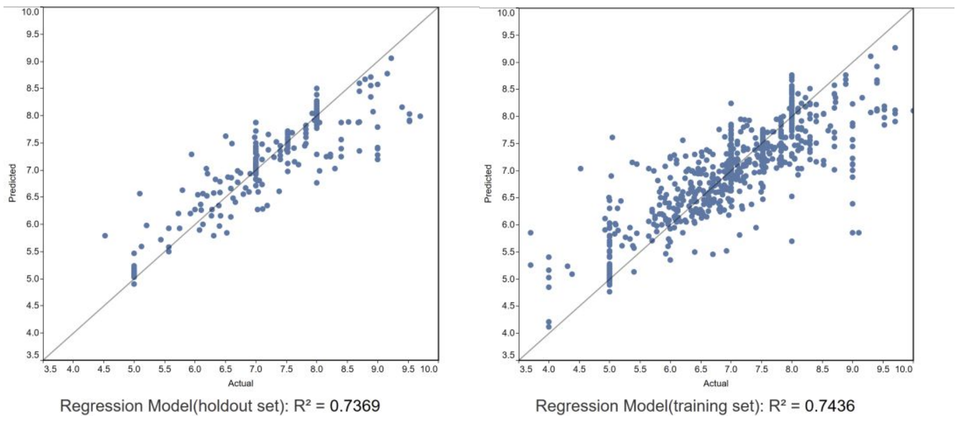

Figure S11. Predicted vs Actual plots for the regression model with RdKit

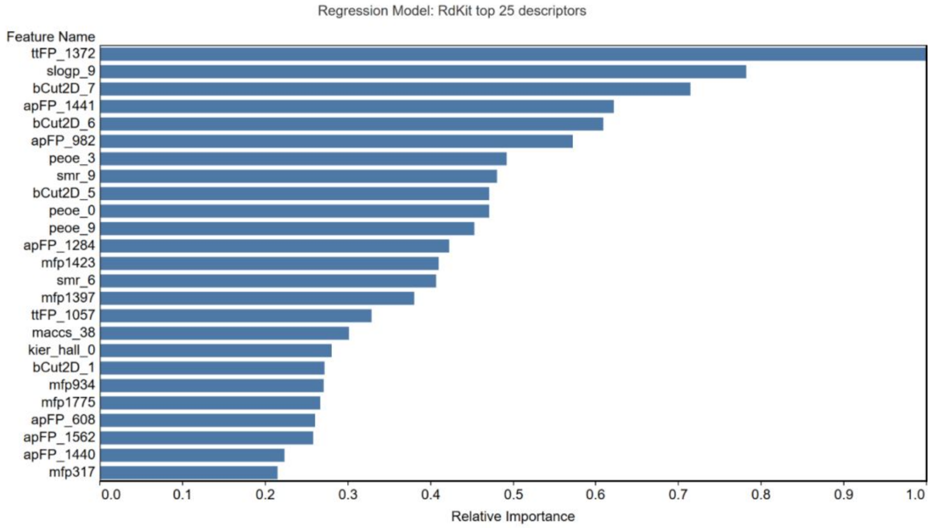

Figure S12. Features impact: top 25 features for the regression model with RdKit

ttFP* - a bit from the topological-torsion fingerprint for a molecule

slogp* - a component of the logP van der Waals surface area (VSA) descriptors

bCut2D* – a component of the 2D BCUT descriptors vector

apFP* – a bit from the atom-pair fingerprint for a molecule

peoe* - a component of the PEOE van der Waals surface area (VSA) descriptors

smr* - a component of the MR van der Waals surface area (VSA) descriptors

mfp* - a bit from the Morgan fingerprint for a molecule

maccs* - a bit from the MACCS fingerprint for a molecule

kier_hall* - the Hall-Kier alpha value for a molecule

1.2. Binary Models

The binary models classify the input data into two categories, for e.g. Active and Inactive. It was built using eXtreme Gradient Boosted Trees Classifier method. Here, we developed the binary model using three sets of molecular descriptors such as CAS fingerprint, RDKit descriptors and fragment-based descriptors to investigate their correlation and contribution towards TBK1 activity. CAS fingerprint – Model: eXtreme Gradient Boosted Trees Classifier (learning rate =0.01)

Statistical measures:

AUC	= 0.9624
F1	= 0.9373
Sensitivity	= 0.9345
Specificity	= 0.8551
Precision	= 0.9401
Accuracy	= 0.9114

To measure the statistical quality of binary models, we used the receiver–operator-characteristic (ROC)-AUC that is a measure of model fit ranging from 0 to 1 and created by plotting the true positive rate against the false positive rate at various threshold settings, with a perfect model having an ROC-AUC of 1.

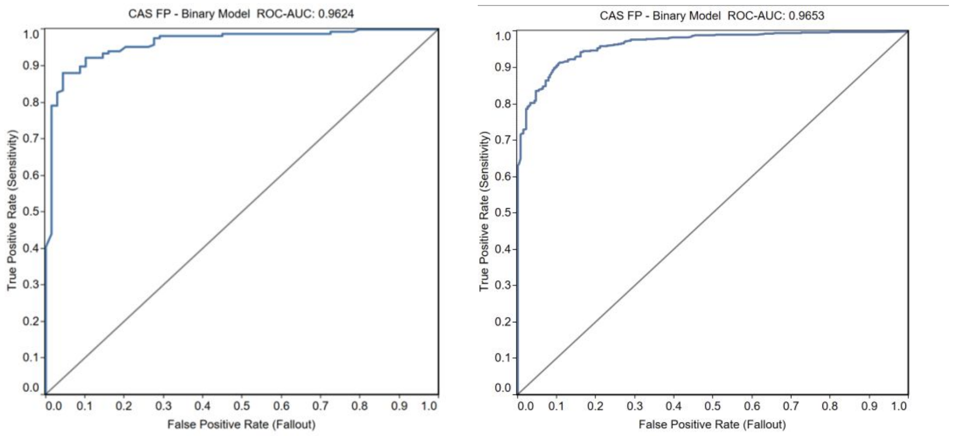

Figure S13. ROC-AUC plots for the binary model with CAS fingerprint

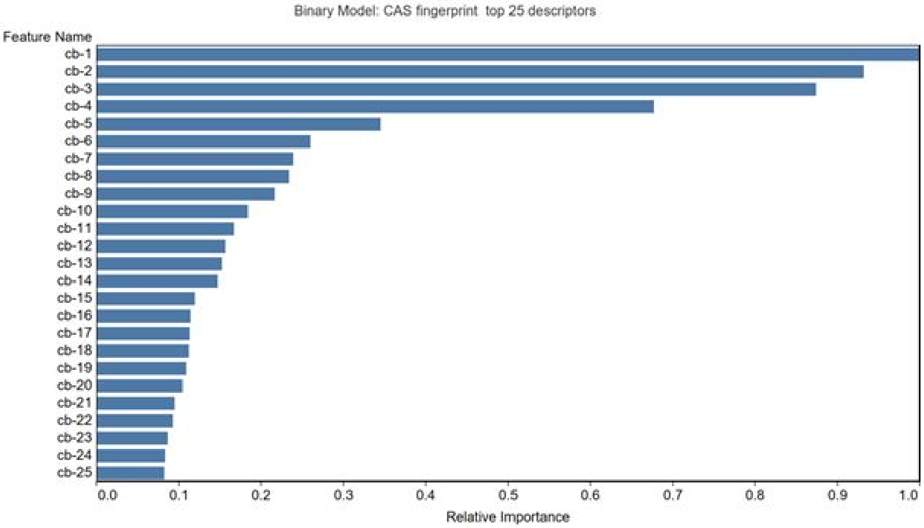

Figure S14. Features impact: top 25 features for the binary model with CAS fingerprint

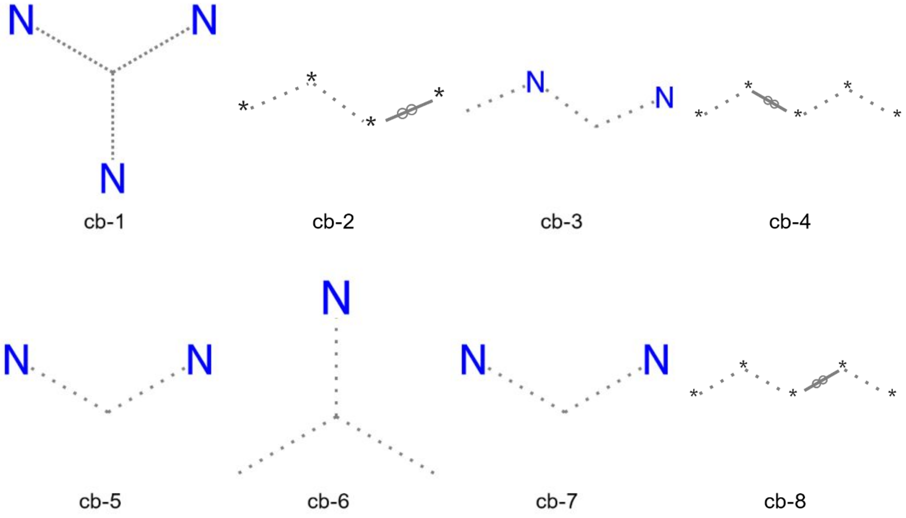

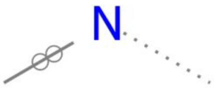

cb-9

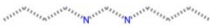

cb-10

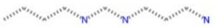

cb-11

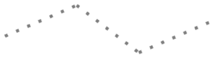

cb-12

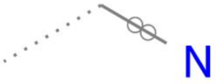

cb-13

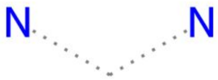

cb-14

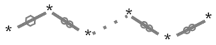

cb-15

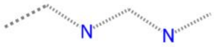

cb-16

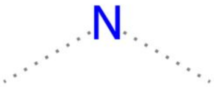

cb-17

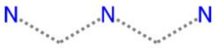

cb-18

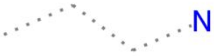

cb-19

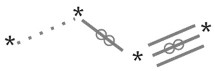

cb-20

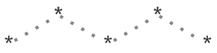

cb-21

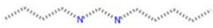

cb-22

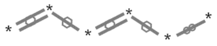

cb-23

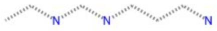

cb-24

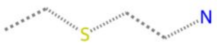

cb-25

Figure S15. Structures of the top 25 features for the binary model with CAS fingerprint

CAS Fragment-functionality analysis – Model: Light Gradient Boosting on ElasticNet Predictions

AUC	= 0.9672
F1	= 0.9535
Sensitivity	= 0.9762
Specificity	= 0.8261
Precision	= 0.9318
Accuracy	= 0.9325

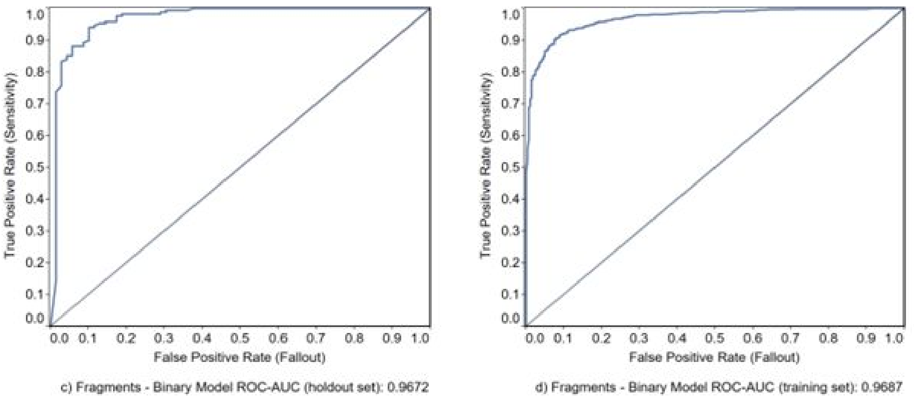

Figure S16. ROC-AUC plot for the binary model with CAS Fragment-functionality

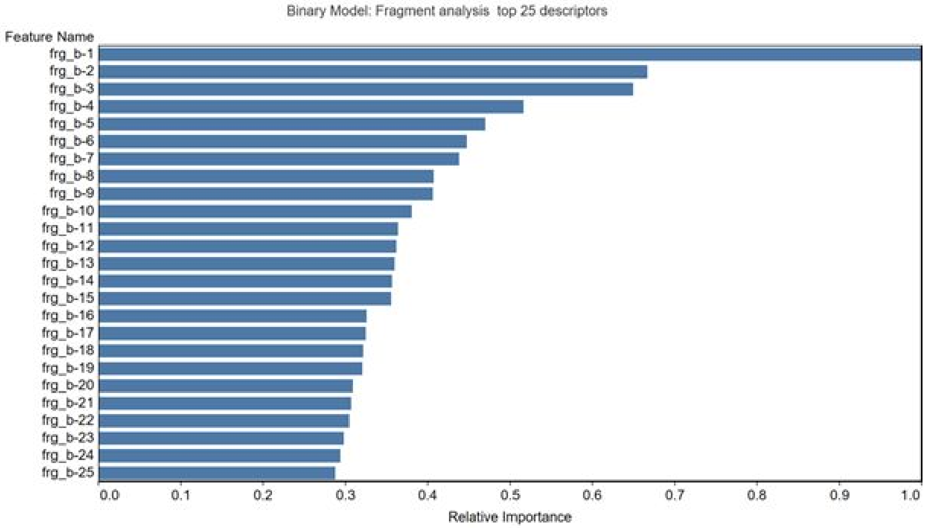

Figure S17. Features impact: top 25 features for the binary model with CAS fragment-functionality

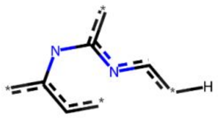

frg_b-1

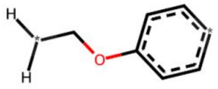

frg_b-2

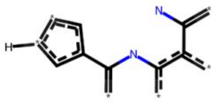

frg_b-3

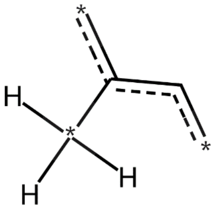

frg_b-4

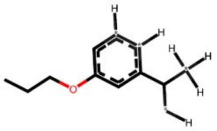

frg_b-5

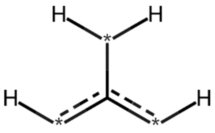

frg_b-6

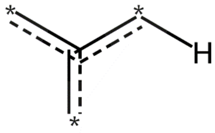

frg_b-7

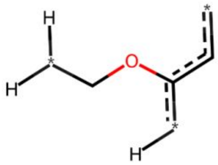

frg_b-8

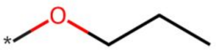

frg_b-9

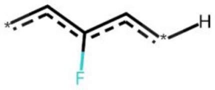

frg_b-10

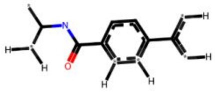

frg_b-11

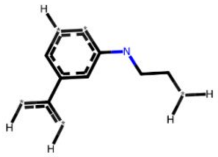

frg_b-12

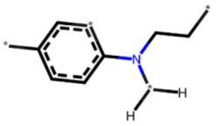

frg_b-13

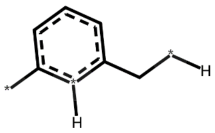

frg_b-14

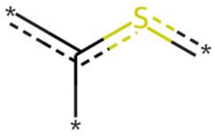

frg_b-15

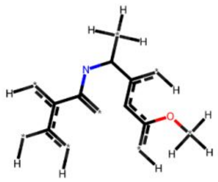

frg_b-16

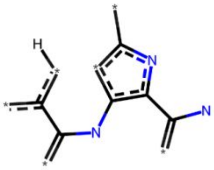

frg_b-17

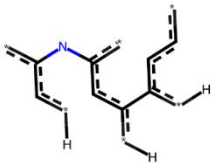

frg_b-18

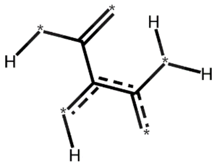

frg_b-19

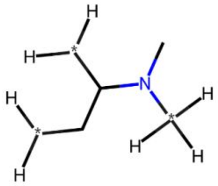

frg_b-20

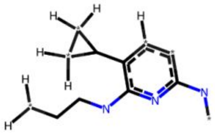

frg_b-21

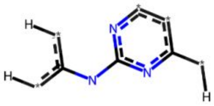

frg_b-22

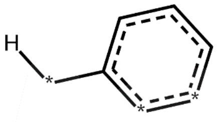

frg_b-23

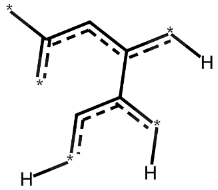

frg_b-24

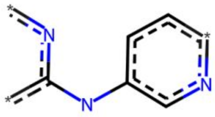

frg_b-25

Figure S18. Structures of the top 25 features for the binary model with CAS Fragment-functionality

RdKit – Model: Gradient Boosted Trees Classifier

AUC	= 0.9714
F1	= 0.9621
Sensitivity	= 0.9821

Specificity	= 0.8551
Precision	= 0.9429
Accuracy	= 0.9451

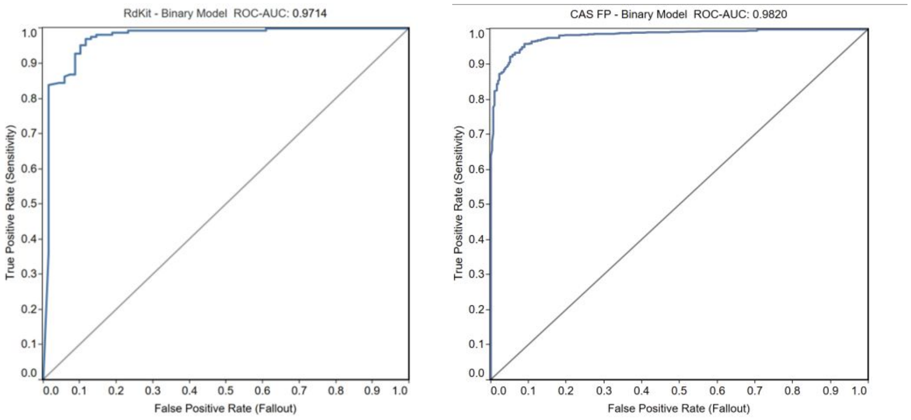

Figure S19. ROC-AUC plots for the binary model with RdKit

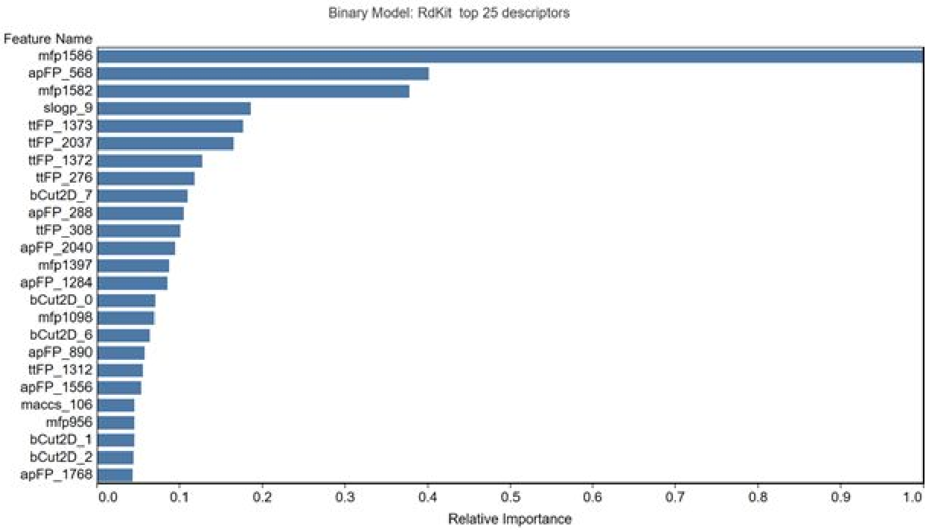

Figure S20. Features impact: top 25 features for the binary model with RdKit

1.3. 3-Category Models

CAS fingerprint – Model:

AUC	= 0.9432
Accuracy	= 0.8565
Balanced Accuracy	= 0.8582
LogLoss	= 0.4256

3-Category Model: CAS FP Confusion Matrix

	Predicted Class		
Actual Class	1	2	3
1	56	6	1
2	7	81	5
3	3	12	66

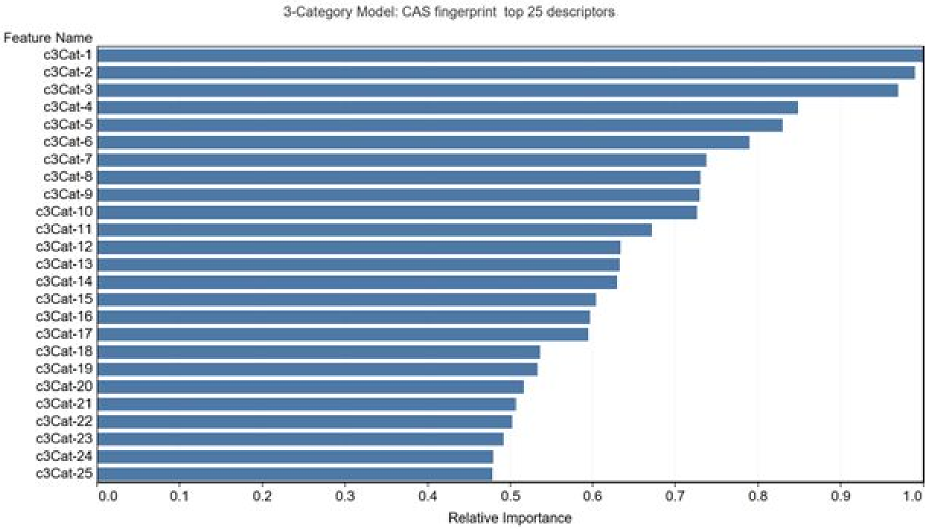

Figure S21. Features impact: top 25 features for the binary model with CAS fingerprint

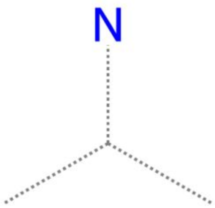

c3Cat-1

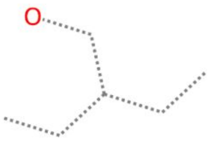

c3Cat-2

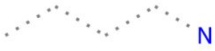

c3Cat-3

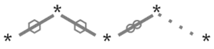

c3Cat-4

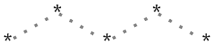

c3Cat-5

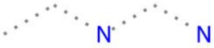

c3Cat-6

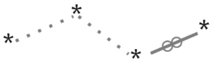

c3Cat-7

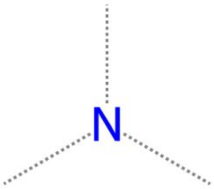

c3Cat-8

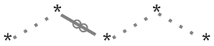

c3Cat-9

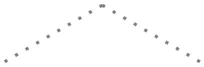

c3Cat-10

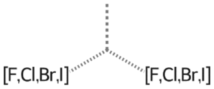

c3Cat-11

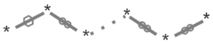

c3Cat-12

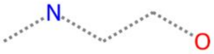

c3Cat-13

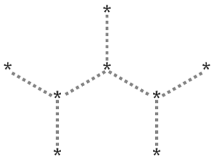

c3Cat-14

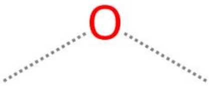

c3Cat-15

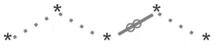

c3Cat-16

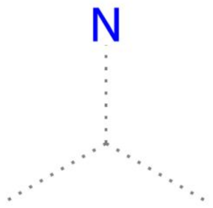

c3Cat-17

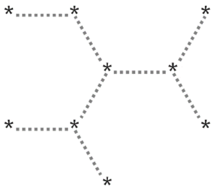

c3Cat-18

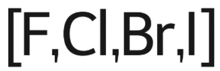

c3Cat-19

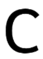

c3Cat-20

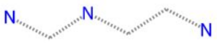

c3Cat-21

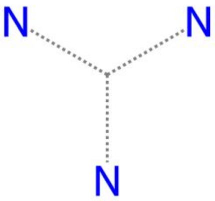

c3Cat-22

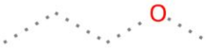

c3Cat-23

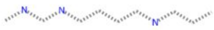

c3Cat-24

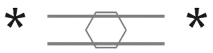

c3Cat-25

Figure S22. Structures of the top 25 features for the 3-category model with CAS fingerprint

CAS Fragment-functionality – Model:

AUC	= 0.9454
Accuracy	= 0.8312
Balanced Accuracy	= 0.8367
LogLoss	= 0.4282

3-Category Model: Fragments
Confusion Matrix

	Predicted Class		
Actual Class	1	2	3
1	56	7	0
2	10	75	8
3	4	11	66

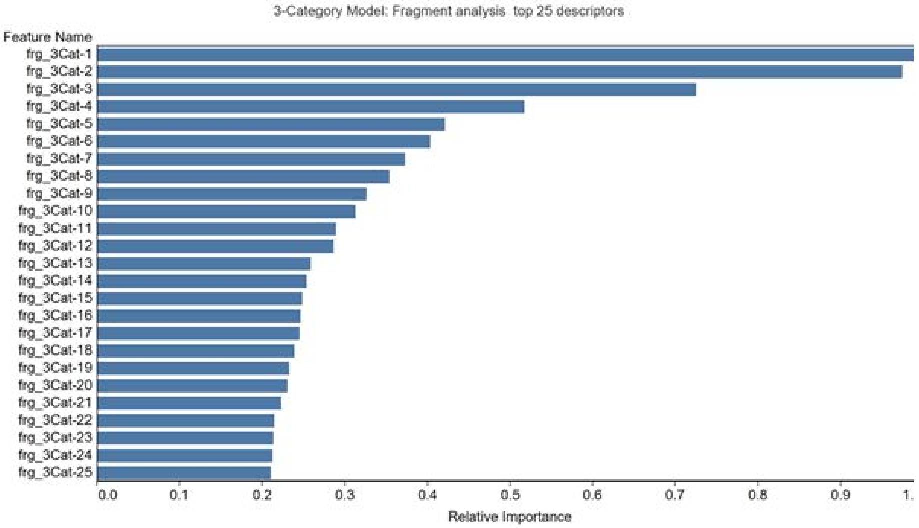

Figure S23. Features impact: top 25 features for the 3-category model with CAS Fragment-functionality

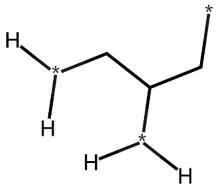

frg_3Cat-1

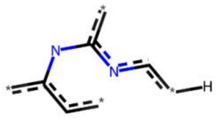

frg_3Cat-2

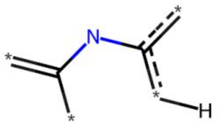

frg_3Cat-3

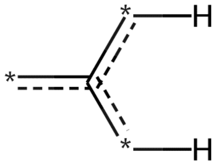

frg_3Cat-4

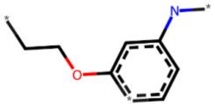

frg_3Cat-5

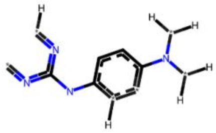

frg_3Cat-6

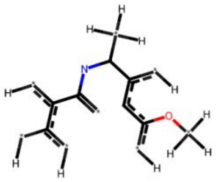

frg_3Cat-7

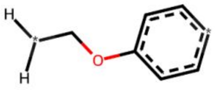

frg_3Cat-8

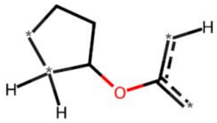

frg_3Cat-9

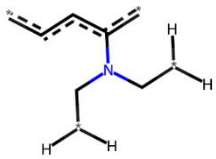

frg_3Cat-10

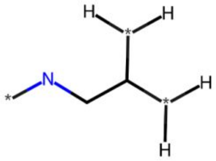

frg_3Cat-11

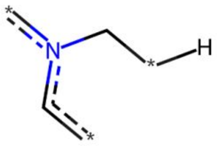

frg_3Cat-12

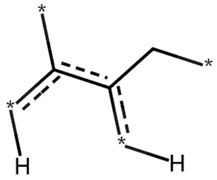

frg_3Cat-13

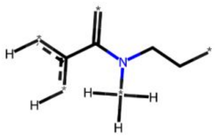

frg_3Cat-14

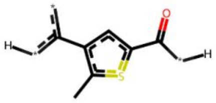

frg_3Cat-15

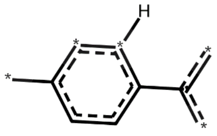

frg_3Cat-16

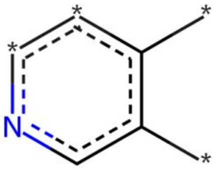

frg_3Cat-17

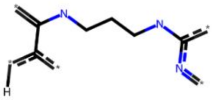

frg_3Cat-18

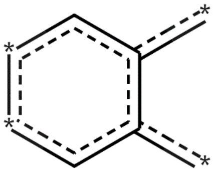

frg_3Cat-19

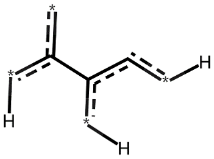

frg_3Cat-20

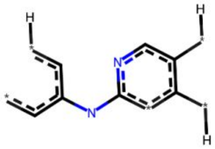

frg_3Cat-21

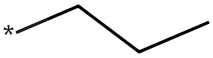

frg_3Cat-22

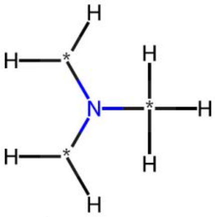

frg_3Cat-23

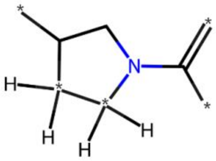

frg_3Cat-24

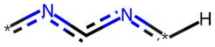

frg_3Cat-25

Figure S24. Structures of the top 25 features for the 3-category model with CAS Fragment-functionality

RdKit – Model:

AUC	= 0.9482
Accuracy	= 0.8523
Balanced Accuracy	= 0.8518
LogLoss	= 0.3919

3-Category Model: RdKit Confusion Matrix

	Predicted Class		
Actual Class	1	2	3
1	54	9	0
2	6	81	6
3	2	12	67

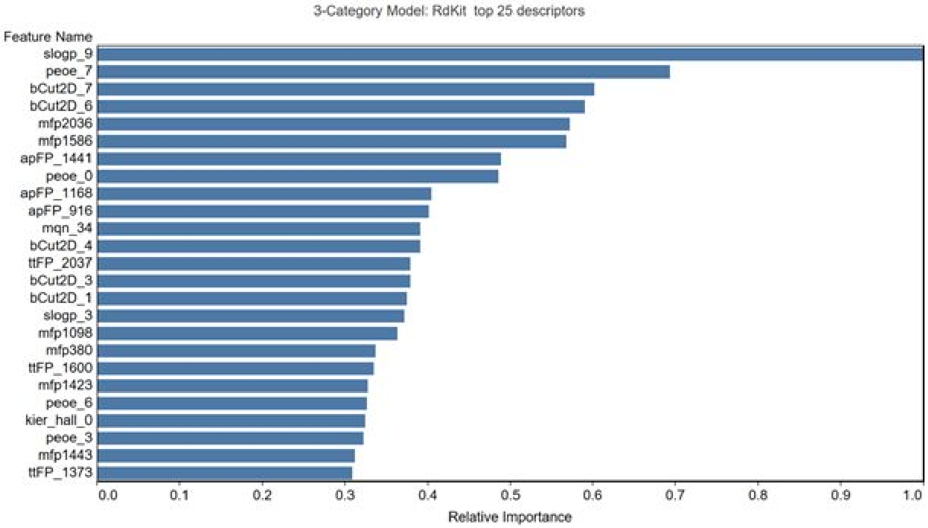

Figure S25. Features impact: top 25 features for the 3-category model with RdKit

The results for the predictions of the external test chemicals using fragment-based models, RdKIT models, and CAS fingerprint models are presented in Supplementary Tables 2, 3, and 4 respectively.

Supplementary Table 2: CAS Fragment-functionality models external predictions.

#	CAS Registry #	pIC50-observed	Binary-observed Breakpoint at pIC50=7	3-Category-observed*	pIC50-predicted	Binary-predicted	3-Category-predicted*
1	68301-99-5	4.26	0	1	5.36	0	1
2	2116443-03-7	4.62	0	1	4.67	0	1
3	2116445-80-6	5.54	0	1	6.22	0	1
4	2116445-76-0	4.00	0	1	5.67	0	1
5	2116445-77-1	4.44	0	1	5.34	0	1
6	2116445-78-2	4.14	0	1	5.33	0	1
7	unknown**	5.35	0	1	5.67	0	1
8	2116445-85-1	4.48	0	1	4.73	0	1
9	70529-18-9	6.40	0	1	5.89	0	1
10	2116445-81-7	5.54	0	1	5.54	0	1
11	2116445-82-8	4.00	0	1	5.80	0	1
12	1056634-68-4	7.24	1	2	7.16	1	3
13	2116443-62-8	6.70	0	1	5.75	0	1
14	2243281-75-4	8.05	1	3	7.80	1	2
15	2243281-77-6	7.70	1	2	7.80	1	2
16	2322365-47-7	8.30	1	3	8.07	0	3
17	81267-65-4	6.02	0	1	5.92	0	1
18	1835675-67-6	8.55	1	3	8.45	0	1
19	2101906-58-3	7.86	1	2	7.76†	1	2
20	1903773-70-5	7.70	1	2	6.61†	0	1
21	2020003-22-7	6.89	0	1	5.21	0	1
					R^2 = 0.69	Accuracy=0.86	Accuracy=0.81

Category 1 (pIC50<7); Category 2 (pIC50<8); Category 3 (pIC50>=8).

**SMILES: CC(C)C1C=CC(=C2C=1)OC3N=C(N)C(C(=O)OCCN(C)C)=CC=3C2=O

†Warning: The production might be incorrect as the chemical lies outside of the applicability domain

Supplementary Table 3: RdKit models external predictions

#	CAS Registry #	pIC50-observed	Binary-observed Breakpoint at pIC50=7	3-Category-observed*	pIC50-predicted	Binary-predicted	3-Category-predicted*
1	68301-99-5	4.26	0	1	5.93	0	1
2	2116443-03-7	4.62	0	1	6.06	0	1
3	2116445-80-6	5.54	0	1	6.52	0	1
4	2116445-76-0	4.00	0	1	6.05	0	1
5	2116445-77-1	4.44	0	1	6.16	0	1
6	2116445-78-2	4.14	0	1	6.01	0	1
7	unknown**	5.35	0	1	6.14	0	1
8	2116445-85-1	4.48	0	1	5.86	0	1
9	70529-18-9	6.40	0	1	6.00	0	1
10	2116445-81-7	5.54	0	1	5.82	0	1
11	2116445-82-8	4.00	0	1	6.11	0	1
12	1056634-68-4	7.24	1	2	7.31	1	3
13	2116443-62-8	6.70	0	1	5.92	0	1
14	2243281-75-4	8.05	1	3	7.61	1	2
15	2243281-77-6	7.70	1	2	7.61	1	2
16	2322365-47-7	8.30	1	3	7.82	1	3
17	81267-65-4	6.02	0	1	5.86	0	1
18	1835675-67-6	8.55	1	3	6.65	0	1
19	2101906-58-3	7.86	1	2	6.13	0	1
20	1903773-70-5	7.70	1	2	7.03	0	1
21	2020003-22-7	6.89	0	1	5.59	0	1
					R^2 = 0.33	Accuracy=0.86	Accuracy=0.76

*Category 1 (pIC50<7); Category 2 (pIC50<8); Category 3 (pIC50>=8).

**SMILES: CC(C)C1C=CC(=C2C=1)OC3N=C(N)C(C(=O)OCCN(C)C)=CC=3C2=O

Supplementary Table 4: CAS fingerprint models external predictions

#	CAS Registry #	pIC50-observed	Binary-observed Breakpoint at pIC50=7	3-Category-observed*	pIC50-predicted	Binary-predicted	3-Category-predicted*
1	68301-99-5	4.26	0	1	6.04	0	1
2	2116443-03-7	4.62	0	1	6.04	0	1
3	2116445-80-6	5.54	0	1	6.21	0	1
4	2116445-76-0	4.00	0	1	6.23	0	1
5	2116445-77-1	4.44	0	1	6.47	0	1
6	2116445-78-2	4.14	0	1	6.54	0	1
7	unknown**	5.35	0	1	6.01	0	1
8	2116445-85-1	4.48	0	1	6.04	0	1
9	70529-18-9	6.40	0	1	6.22	0	1
10	2116445-81-7	5.54	0	1	6.09	0	1
11	2116445-82-8	4.00	0	1	6.08	0	1
12	1056634-68-4	7.24	1	2	5.85	1	3
13	2116443-62-8	6.70	0	1	6.07	0	1
14	2243281-75-4	8.05	1	3	7.65	1	2
15	2243281-77-6	7.70	1	2	7.65	1	2
16	2322365-47-7	8.30	1	3	8.10	1	3
17	81267-65-4	6.02	0	1	5.52	0	1
18	1835675-67-6	8.55	1	3	5.18	0	1
19	2101906-58-3	7.86	1	2	6.51	0	1
20	1903773-70-5	7.70	1	2	6.09	0	1
21	2020003-22-7	6.89	0	1	5.25	0	1
					R^2 = 0.01	Accuracy=0.86	Accuracy=0.76

*Category 1 (pIC50<7); Category 2 (pIC50<8); Category 3 (pIC50>=8).

**SMILES: CC(C)C1C=CC(=C2C=1)OC3N=C(N)C(C(=O)OCCN(C)C)=CC=3C2=O

Supplementary Table 5: Best predicted TBK1 inhibitors

ID	Residuals
1414935800	0.01
2376439120	0.03
1333108578	0.04
2376439073	0.07
1414935606	0.07
2376439222	0.08
862722916	0.08
2376439200	0.1
2376439313	0.11
2376439095	0.14
1333108216	0.15
2116442078	0.19
2376439084	0.19
152121476	0.2
2376439346	0.24
1414935775	0.29
1414949975	0.29
1365645313	0.37
285983484	0.47
2116442783	0.48

Existing TBK1 inhibitors:

Name (CAS RN)	Structure	Reported IC50
MRT67307 (1190378-57-4)	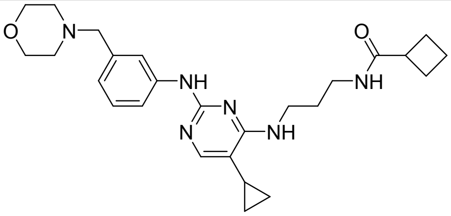	19nM

BX795 (702675-74-9)	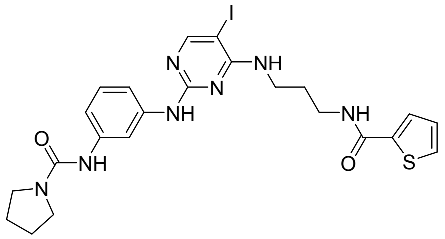	0.006 µM
Amlexanox (68302-57-8)	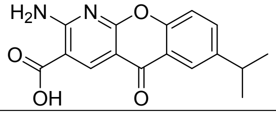	0.8 µM
Amlexanox Analog 1 (70529-18-9)	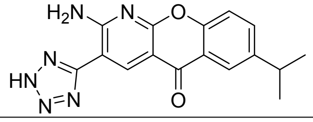	400nM
Amlexanox Analog 2 (2116442-11-4)	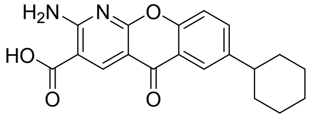	200nM
Amlexanox Analog 3 (2116442-78-3)	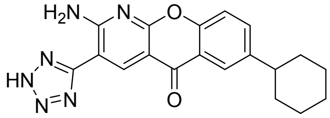	1.9uM
Compound II (1381930-17-1)	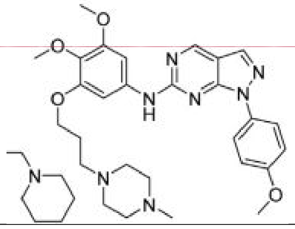	13nM
BAY-985 (2409479-29-2)	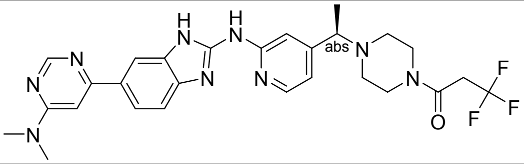	2 nM (IC50, low ATP)<br><br>30 nM (IC50, high ATP)

Commented [KI1]: @Ralhan, Krittika Z - this is the structure without the piperidinyl component at the bottom left. Can you please point me in the direction of the publication that describes this structure? Thanks!

Commented [RZ2R1]: @Iyer, Kavita TANK-binding kinase 1 (TBK1): An emerging therapeutic target for drug discovery - ScienceDirect please see this one.

AZ3102909 (2322365-47-7)	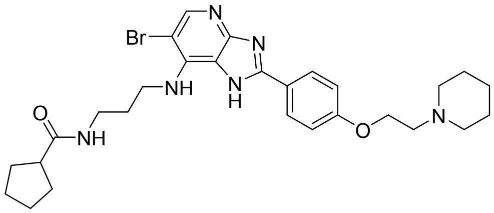	5nM
CYT387 (1056634-68-4)	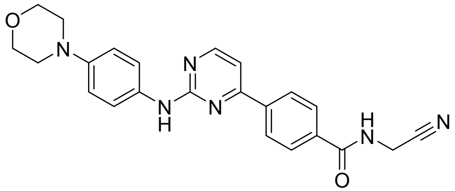	58nM
GSK8612 (2361659-62-1)	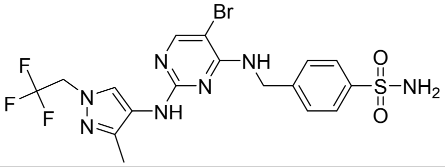	pIC50- 6.8
